# The purinergic signaling interfaces in breast cancer angiogenesis

**DOI:** 10.1007/s11302-025-10119-1

**Published:** 2026-01-23

**Authors:** Fernanda Cardoso da Silva, Jeferson Stabile, Cristina Ribas Fürstenau, Thaise Gonçalves Araújo

**Affiliations:** 1 Laboratory of Genetics and Biotechnology, Institute of Biotechnology, Federal University of Uberlândia, Patos de Minas, MG Brazil; 2 Laboratory of Vascular Biochemistry, Center for Natural and Human Sciences, Federal University of ABC, Santo André, SP Brazil

**Keywords:** Adenosine, ATP, Anti-angiogenic, Tumor angiogenesis, Breast cancer

## Abstract

Cancer is a group of diseases characterized by disordered cell proliferation and loss of tissue architecture. Breast cancer (BC) is the most common and lethal cancer among women, standing out for its molecular, histological and pathological heterogeneity. The BC tumor microenvironment (TME) is a complex ecosystem comprising transformed cells and a multitude of non-tumor cells, embedded in an altered extracellular matrix. Endothelial cells are present, driving angiogenesis, a relevant hallmark that ensures nutrition and oxygenation through the formation of new blood vessels. During this process, a complex network of molecules is released by tumor and endothelial cells, such as Vascular Endothelial Growth Factor (VEGF), that, in turn, induce cancer progression, diffusion, and metastasis. Purinergic signaling also regulates the functioning of endothelial cells involving the action of purines (ATP, ADP, UTP, UDP and adenosine) as signaling in purinergic receptors, with their concentration modulated by enzymes known as ectonucleotidases. This review aims to explore the contribution of purinergic signaling to BC angiogenesis, highlighting potential therapeutic targets currently under scientific focus. In general, the TME presents overexpression of ATP and adenosine, which stimulate endothelial cells through purinergic receptors. This stimulus promotes the formation of new vessels, mainly via the release of VEGF. Thus, purinergic signaling emerges as a central mechanism in BC angiogenesis, with potential to be explored in the development of antitumor therapies.

## Introduction

Cancer is a complex and multifactorial disease primarily characterized by losing control over the cell cycle. According to the Global Cancer Observatory, cancer was the second most common disease in the world population in 2022, with 2,296,840 registered cases, and more than 666 thousand deaths [1]. Breast cancer (BC) is one of the most prevalent malignancies and a leading cause of cancer-related mortality among women worldwide [2].

Despite significant advances in early detection and therapeutic strategies, BC remains a daunting public health challenge due to its complex biology and high rates of recurrence and metastasis. The main concerns are drug resistance and the activation of alternative pathways that enable cancer survival in stressful environments. Therefore, understanding the intricate mechanisms underlying BC progression is critical for developing more effective and targeted interventions [3]. The tumor microenvironment (TME) is essential in BC progression, influencing tumor growth, invasion, and metastasis. In fact, TME produces many factors that promote the formation of new blood vessels, a process known as angiogenesis. Angiogenesis, in turn, supports tumor expansion and dissemination by ensuring an adequate supply of nutrients and oxygen, forming a malignant tumor growth-promoting cycle [4]. Angiogenesis in BC have been a focus of intense research, revealing a multitude of signaling pathways that orchestrate this cancer hallmark [5, 6].

Purinergic signaling has garnered increasing attention in recent years. This intricate communication system, mediated by extracellular nucleotides, nucleosides, and their associated receptors, is critical in regulating numerous physiological and pathological processes, including inflammation, immune response, and tissue remodeling [7]. Ectonucleotidases, enzymes responsible for the hydrolysis of extracellular nucleotides, further modulate this signaling network, adding complexity to its role in cancer biology [8]. In the context of BC, purinergic signaling modulates tumor cell proliferation, immune evasion, and vascular remodeling [9]. Recent evidence suggests that purinergic signaling interacts closely with angiogenic pathways, influencing the formation and function of the tumor vasculature, inhibiting apoptosis, increasing immune evasion through the downregulation cytotoxic T-cells, and the generation of tolerogenic leukocytes [10]. This interplay represents a novel axis of investigation with significant implications for understanding BC progression and identifying therapeutic targets.

This review aims to provide a comprehensive overview of the epidemiology and molecular aspects of BC, with a particular focus on the role of TME and angiogenesis. It explores the molecular mechanisms underlying angiogenesis, delves into the emerging significance of purinergic signaling in cancer and vascular biology, and examines the interaction between purinergic signaling and angiogenesis in BC. Finally, the review highlights future perspectives and discusses therapeutic innovations that leverage these insights to improve BC management.

## Epidemiology and molecular aspects of breast cancer

BC is the second most common cancer, accounting for 11.5% of new cases in both sexes in 2022 [11]. Worldwide, there are differences in epidemiological data according to the human development index (HDI) of the countries. Those with higher HDI are the most affected, such as the United States and European countries. However, mortality is more significant in those with lower HDI, since the population have limited access to early diagnosis and adequate treatment [12].

A study developed by Xu and collaborators pointed that in 1990, 876,990 cases of BC were diagnosed, while in 2019 there were 2,002,350, indicating an increase in both incidence and diagnostic effectiveness. As for mortality, in 2019, approximately 700,000 deaths were recorded, significantly higher than in 1990, about 380,000 deaths [13]. However, since 1989, mortality rate has decreased due to the combination of improvements in diagnostic methods, early detection and greater availability of therapies [14]. Despite the numbers, some specific groups remain at risk. Data indicate that metabolic factors, such as high blood glucose levels and overweight/obesity, contribute significantly to mortality from BC. In addition, behavioral factors, such as a sedentary lifestyle, and changes in eating habits also aggravate this scenario [13]. Ethnicity and race are also risk factors for BC. Incidence and mortality differ between white and black women. Black women have an approximately 40% higher risk of dying from BC compared to white women revealing not only genetic differences, but also disparities in access to medical care [15, 16].

Diagnostic and treatment strategies have evolved over the last 25 years, but the forecasts are still alarming, pointing to a growing increase of cases over the coming decades. By 2040, the number of new diagnostics is expected to reach 3 million per year, while the deaths could reach 1 million per year. This reality highlights the need for a more in-depth understanding of BC biology. In fact, BC is a heterogenous disease from a histological and, mainly, molecular point of view [13, 16]. In 2013, the International BC Conference in St. Gallen defined the following BC molecular subtypes: luminal A, luminal B, human epidermal growth factor receptor-type 2(HER2)-enriched (HER2E), and triple-negative (TNBC), based on immunohistochemical classification for estrogen, progesterone, and HER2 receptors (the last confirmed by fluorescence in situ hybridization). New classification systems also consider Ki-67, a nuclear antigen expressed in cells with high proliferation index, to differentiate luminal A to luminal B cancers [17–22].

Luminal BC, subtypes A and B, are positive for hormone receptors and represents the most frequent cases [23]. The luminal A has a better prognosis and low risk of metastases affecting half of patients with BC. In contrast, the luminal B exhibits a less favorable prognosis, high histological grade, and high cell proliferation compared to luminal A, accounting for 15% of BC diagnoses [17]. Regarding Ki-67, luminal A expresses lower Ki-67 levels (< 20%), while luminal B presents Ki-67 ≥ 20% [24, 25]. First-line therapy for Luminal BC is hormonal therapy, which aims to inhibit hormone receptors or the aromatase enzyme, responsible for converting androstenedione and testosterone into estrogen. The main agents include selective estrogen receptor modulators, such as tamoxifen, and aromatase inhibitors (anastrozole, letrozole and exemestane). In patients with HER2-positive luminal B (luminal-HER2) BC, anti-HER2 therapies can also be used. In cases of greater tumor aggressiveness or resistance to hormonal therapy, chemotherapy may be adopted [26].

The HER2E BC subtype, in turn, corresponds to 15 to 20% of BC cases, standing out for the high expression of the HER2 oncoprotein, absence of hormone receptors, and intermediate histological grade. In this tumor subtype, metastases are more frequent in brain, bones, liver, and lungs [17, 27]. Among the existing therapies for HER2-positive BC, first-line treatment consists of the use of monoclonal antibodies that target the HER2 receptor. These antibodies block intracellular signaling involved in the PI3K/AKT and MAPK pathways, resulting in the inhibition of cell proliferation and reduced tissue invasion. Furthermore, chemotherapy can be used mainly in cases of metastasis and tissue invasion [28].

Finally, the TNBC subtype does not express any of the aforementioned receptors, being the most heterogeneous group among BC subtypes. Although most TNBCs belong to the basal-like subtype, this overlap is not complete, highlighting the diversity of this group. The TME is highly variable with signaling pathways associated with the immune response, angiogenesis, and metabolic alterations [20, 29, 30]. TNBC is the most aggressive, with a high histological grade and mitotic rate. Another aspect of this subtype is the high propensity for metastasis. TNBC represents between 10 and 20% of BC cases and is treated mainly with chemotherapy, preferably in the neoadjuvant regimen. Other approaches include targeted therapies, such as DNA repair inhibitors, especially poly(ADP-ribose) polymerase (PARP) inhibitors, such as olaparib and talazoparib, indicated for patients with mutations in the BRCA1/2 genes, in which DNA repair deficiency favors synthetic lethality. In addition, immunotherapy with checkpoint inhibitors, such as pembrolizumab and avelumab, has shown benefit, especially in tumors with high programmed death-1 (PD-1) ligand 1 (PD-L1) expression [17, 31].

Table 1 summarizes general information about BC molecular subtypes.

**Table 1:** Breast cancer molecular subtypes: classification, prognosis, and available treatments

Breast Cancer Subtype	Molecular characteristics	Incidence (% of total cases)	Prognosis	Target-therapies*	Available therapies	References
Luminal A	ER + PR + HER2 – Ki-67 < 20%	50%	Better prognosis and low risk of metastases	ER/PR	Estrogen receptor inhibitor (Tamoxifen); Aromatase inhibitors (anastrozole, letrozole, exemestane); Chemotherapy*	[32–35]
Luminal B	ER + PR + HER2 +/– Ki-67 > 20%	15%	Less favorable prognosis than luminal A, high histological grade, and high cell proliferation	ER/PR, HER2	Estrogen receptor inhibitor (Tamoxifen); Aromatase inhibitors (anastrozole, letrozole, exemestane); HER2-targeted therapies (trastuzumab, pertuzumab, and lapatinib); Chemotherapy	[32–34]
HER2E	ER - PR - HER2 +	15–20%	Most frequent metastases in the brain, bones, liver, and lungs	HER2	HER2-targeted therapies (trastuzumab, pertuzumab, lapatinib, and T-DM1); Chemotherapy	[32, 33, 36]
Triple-negative (TNBC)	ER - PR - HER2 -	20%	More aggressive, with a high histological grade, elevated mitotic index, and increased metastatic potential	Inhibitors of DNA repair enzymes in patients with BRCA1/2 mutation Checkpoint inhibitors	Chemotherapy; Immunotherapy checkpoint inhibitors; (atezolizumab and pembrolizumab)* and PARP inhibitors (olaparib and talazoparib).*	[32, 33, 37]

(+) indicates presence, (-) indicates absence, (±) indicates that can be or cannot be present. ER: estrogen receptor; PR: progesterone receptor; HER2: human epidermal growth factor receptor-type 2; BRCA1/2: BRCA1/2 DNA repair associated; PARP: poli(ADP-ribose) polymerase. * These therapies are not eligible for all patients

## Tumor microenvironment and breast cancer progression

The heterogeneity of BC may reflect a specific cellular, molecular and functional organization, which involves not only tumor cells, but also immune cells, blood vessels, extracellular matrix (ECM) and different biomolecules. This TME directly influences the development and progression of the disease, modulating the intrinsic behavior of tumor cells and may favor or block processes such as proliferation, invasion, angiogenesis and therapeutic response. Therefore, the modulation of TME is essential for the advancement of effective treatment strategies [38, 39].

**Fig. 1:**
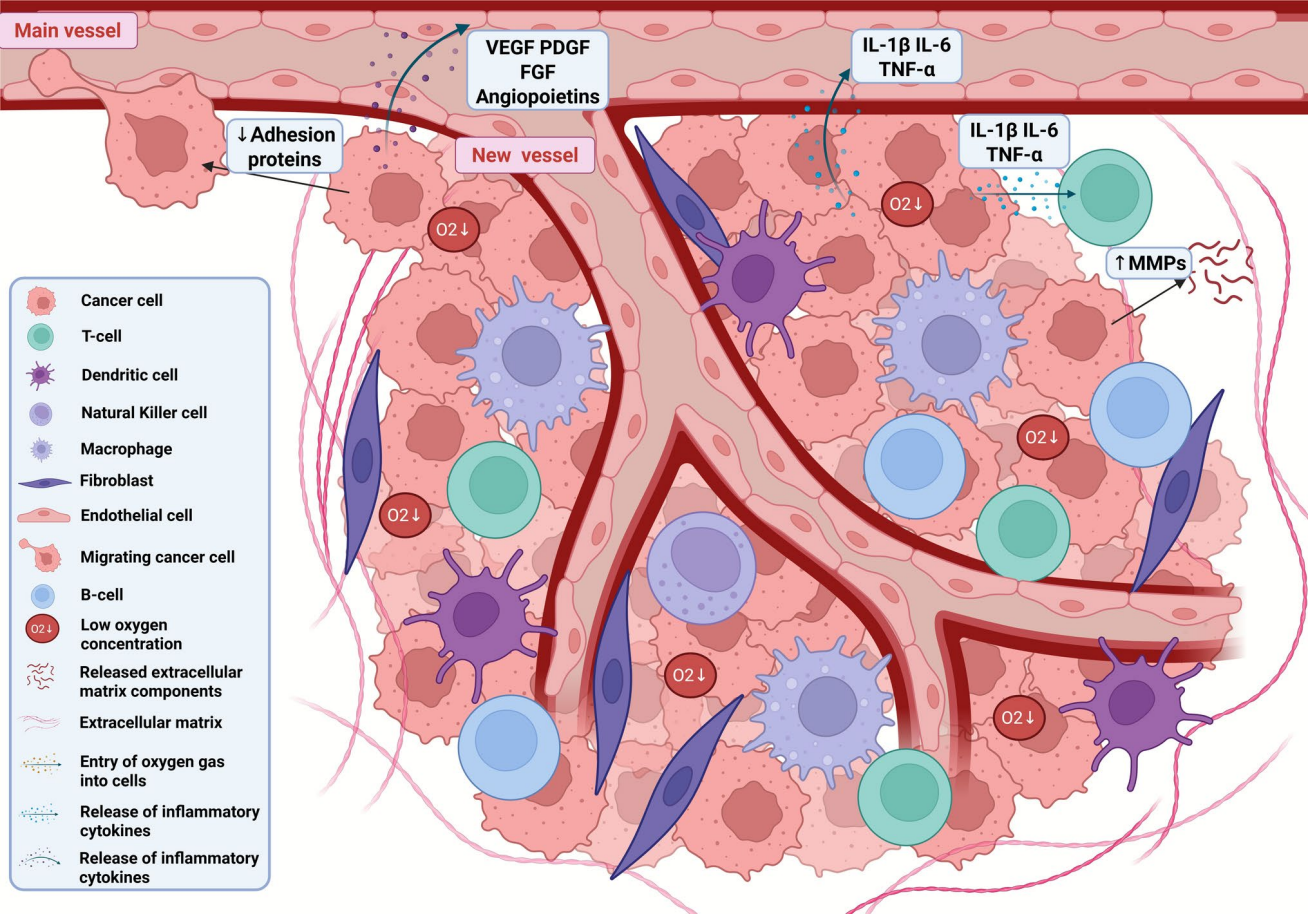
The tumor microenvironment (TME) comprises not only tumor cells but also a diverse array of stromal and immune cells, including T cells, B cells, natural killer cells, dendritic cells, macrophages, and fibroblasts. It is also marked by hypoxia, increased expression of matrix metalloproteinases, reduced cell–cell adhesion, and enhanced angiogenesis. The latter is driven by tumor-derived pro-angiogenic factors such as VEGF, PDGF, angiopoietins, IL-1β, IL-6, and TNF-α. FGF: Fibroblast Growth Factor, IL-1β: Interleukin-1 Beta; IL-6: Interleukin-6; MMPs: Matrix Metalloproteinases; PDGF: Platelet-Derived Growth Factor; TNF-α: Tumor Necrosis Factor Alpha; VEGF: Vascular Endothelial Growth Factor. Created in https://BioRender.com

Firstly, several cellular components and molecules are present (Fig. 1), including lymphocytes, neutrophils, mast cells, pericytes, cancer-associated fibroblasts (CAFs), mesenchymal stem cells, and immunological components [40, 41]. Inflammation in the TME results from a complex interaction between the immune system and cancer cells [42]. Throughout tumor progression, immunological response acts as a barrier to the development and progression of the disease. However, it can also favor metastatic dissemination, highlighting a dual influence on tumor behavior. In the early stages of BC, for example, cytotoxic T-cells and natural killer (NK) cells eliminate transformed cells, limiting tumor growth [42]. As the disease progresses, inflammatory processes can favor progression and metastasis. In this context, leukocyte infiltration stands out, especially tumor-associated macrophages (TAM). These cells increase the production of pro-inflammatory cytokines, such as tumor necrosis factor (TNF), interleukin 1 (IL-1), colony stimulating factor 1 (CSF-1), interleukin 6 (IL-6), C–C motif chemokine ligand 2 (CCL2), and cyclooxygenase-2 (COX-2). These mediators intensify cell proliferation and tumor invasion, remodel ECM and induce angiogenesis [43–45].

The inflammatory profile varies according to BC subtypes. HER2E and TNBC present higher mutational load and therefore tend to be more immunogenic than luminal tumors [46]. TNBC expresses high levels of pro-inflammatory cytokines TNF-α and IL-1β. In contrast, this subtype usually activate PD-L1 and/or TGF-β (Transforming Growth Factor Beta) immunosuppressive pathways that restore tumor aggressiveness [47].

The ECM is also remodeled in BC [48]. Higher collagen density in the TME is associated with greater BC stiffness and worse prognosis [49]. These proteins create an important dynamic coating that favors cell migration and proliferation, associated to overexpression of matrix metalloproteinases [50].

In addition to the immune response and ECM remodeling, the vascular network and the dysregulated metabolism are also important. This dysregulation is caused by a significant difference between the low availability of oxygen and its rapid consumption in energy processes, leading to a state of hypoxia. The hypoxic state is marked by an oxygen pressure of less than 10 mmHg, which is significantly low compared to normal tissue that reaches 40–60 mmHg. In this scenario gene expression and energy metabolism are modulated in such a way that even in the presence of oxygen, tumor cells convert glucose into lactate, prioritizing anaerobic metabolism, which is called the Warburg effect [51]. However, transformed cells can survive in a hypoxic environment, regulating the expression of glucose transporters (GLUT1 and GLUT3), lactate dehydrogenase A (LDHA) and pyruvate kinase 2 (PKM2) to increase glucose uptake [4, 52]. Interestingly, hypoxia is correlated with tumor immunosuppression in BC, especially in TNBC and HER2E, in which less cytotoxic T-cells and NK infiltration is observed after activation of the HIF-1α (Hypoxia-Inducible Factor 1 Alpha) pathway [53]. Adhesion molecules are also altered, such as E-cadherin, N-cadherin, vimentin, and matrix metalloproteinases [4, 52]. Finaly, pro-angiogenic molecules are produced, including vascular endothelial growth factor (VEGF), platelet-derived growth factor (PDGF), fibroblast growth factor (FGF), and angiopoietins [54] (Fig. 1).

Angiogenesis, defined by the formation of new vessels, occurs to overcome a hypoxic and acidic microenvironment, restore the supply of nutrients and oxygen, and remove metabolic residues [55–57]. Hypoxia may induce angiogenesis via PHD2 (prolyl hydroxylase domain 2) inactivation, which leads to the stabilization and activation of HIF-1α pathways [58]. In this process, two cell types play a central role, modulating and giving atypical characteristics to preformed vessels: endothelial cells and pericytes. Endothelial cells form a monolayer that lines the vessels internally, while pericytes surround the vessels externally, providing structural support [59]. In BC, especially in TNBC, angiogenesis is a hallmark of aggressiveness, higher metastasis rate and worse prognosis [47].

## Molecular mechanisms of angiogenesis in breast cancer

Folkman’s theory, proposed in 1971, states that angiogenesis is essential for the maintenance and growth of solid tumors from 1–2 mm^3^. Even decades later, this theory remains widespread and accepted, given that antiangiogenic strategies have been explored and indicated as effective in the treatment of solid tumors, especially in BC [60]. Angiogenesis is considered a cancer hallmark because it allows the rapid progression, growth and dissemination of transformed cells [5].

**Fig. 2:**
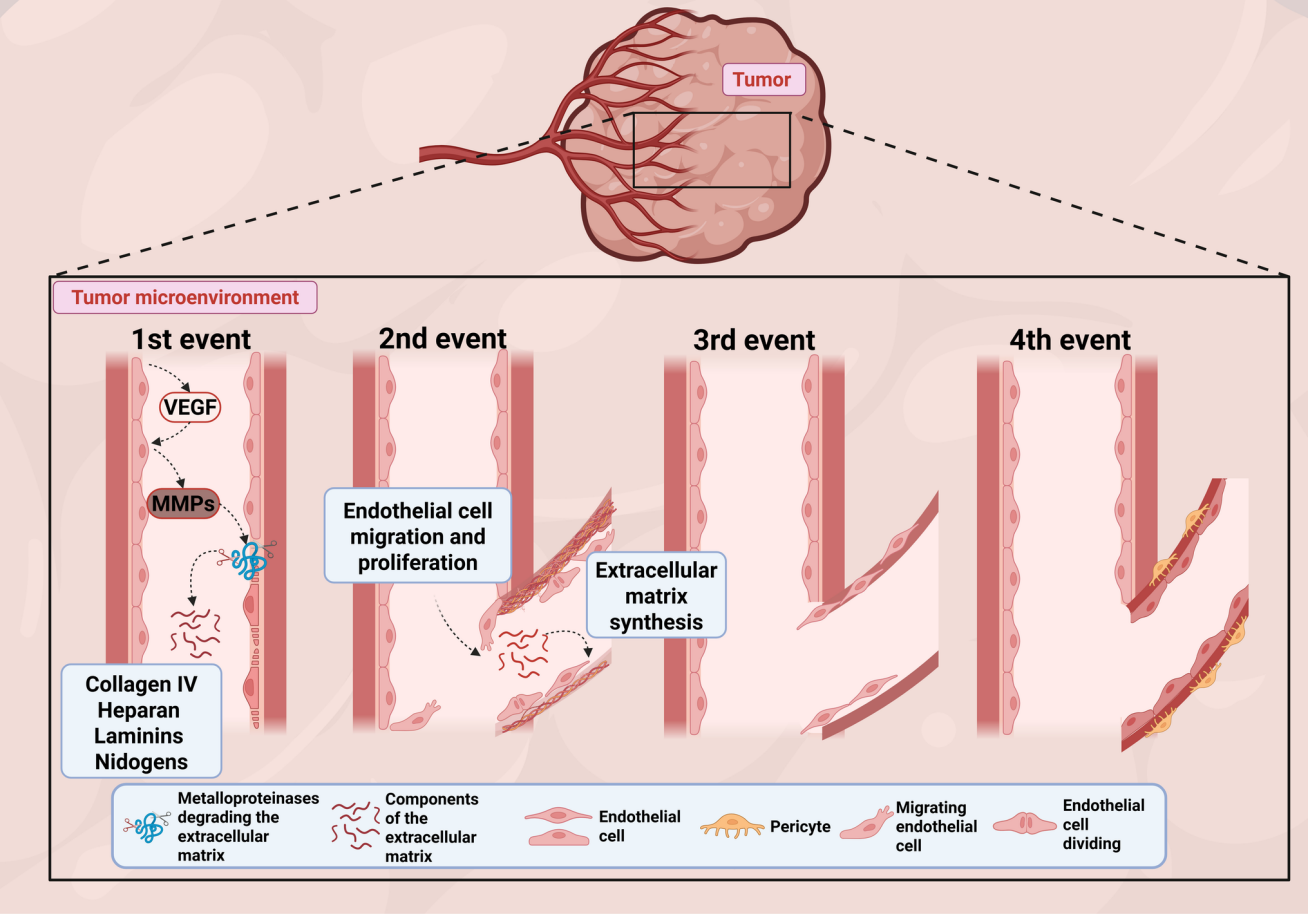
The general process of angiogenesis within the tumor microenvironment. Four events are described: 1) degradation of the basement membrane of existing blood vessels, triggered by VEGF release; 2) development of a provisional extracellular matrix and migration/proliferation of endothelial cells; 3) formation of the vascular lumen, creating vascular tubes; and 4) migration of pericytes and formation of the basement membrane of new blood vessels. MMPs: Matrix Metalloproteinases; VEGF: Vascular Endothelial Growth Factor. Created in https://BioRender.com

During vascular angiogenesis, irregular endothelium and anomalous deposition of the basement membrane are present. Such peculiar characteristics lead to the formation of vessels with naturally increased caliber, tortuous and disorganized, with high permeability and poor perfusion, compromising circulatory function [58]. Four molecular events are associated with this process (Fig. 2). In the first one, VEGF release triggers the degradation of the basement membrane of pre-existing blood vessels, a membrane formed by type IV collagen (collagen IV), heparan sulfate proteoglycan, laminins, and nidogens [58]. In the second stage, a provisional ECM constituted by fibrin, vitronectin, and fibronectin is formed, accompanied by the migration and proliferation of endothelial cells in this matrix. The third event is vascular lumen formation, creating true vascular tubes. Finally, pericytes migration occurs, giving rise to a basal membrane characteristic of blood vessels [54, 61].

The process that promotes angiogenesis in BC is complex and involves different signaling pathways, changes in gene expression, and secretion of biomolecules. In general, pro-angiogenic biomolecules are released; gene expression is regulated; cell adhesion proteins are modulated; and a different profile of exosomes is secreted. These mechanisms contribute to the development of a characteristic vascular network [62].

BC cells can promote angiogenesis by directly releasing VEGF, FGF, interleukins (mainly IL-1β and IL-6), TGF-β, and PDGF through HIF pathway, which forms disorganized and leaky microvessels in TME [6, 63, 64]. Consequently, intratumoral hypoxia is amplified, and facilitates metastatic spread, and suppresses antitumor immune responses. The coordinated interaction between classical angiogenic inducers, angiogenic receptors, and ECM remodeling is crucial [50].

The HIF pathway is considered a central mechanism in angiogenesis, acting as an important transcription factor. However, new mechanisms are being described. The non-coding RNA RAB11B-AS1, for example, controls VEGF expression. Non-coding RNAs are RNAs that are not translated into proteins, including microRNA (miRNA), circular RNA (circRNA), PIWI-interacting RNA (piRNA), and long ncRNA (lncRNA) [65]. In the nucleus, the lncRNA RAB11B-AS1 facilitates the recruitment of RNA polymerase increasing VEGF transcription [6]. Different miRNAs have also been associated to BC angiogenesis such as: Let-7f (which targets TSP-1, Thrombospondin-1), miR-10b (which targets NOTCH1, Notch receptor 1) and miR-126 (which regulates Ang1, Angiopoietin-1), with pro-angiogenic properties. On the other hand, the miRNAs miR-145 (targeting VEGF-A, Vascular Endothelial Growth Factor A) and miR-195 (targeting VEGF) are classified as anti-angiogenic [66]. Hedgehog signaling pathway is also important, since it regulates the expression of immune system proteins and CD8 + T lymphocyte infiltration, controlling angiogenesis [67].

**Fig. 3:**
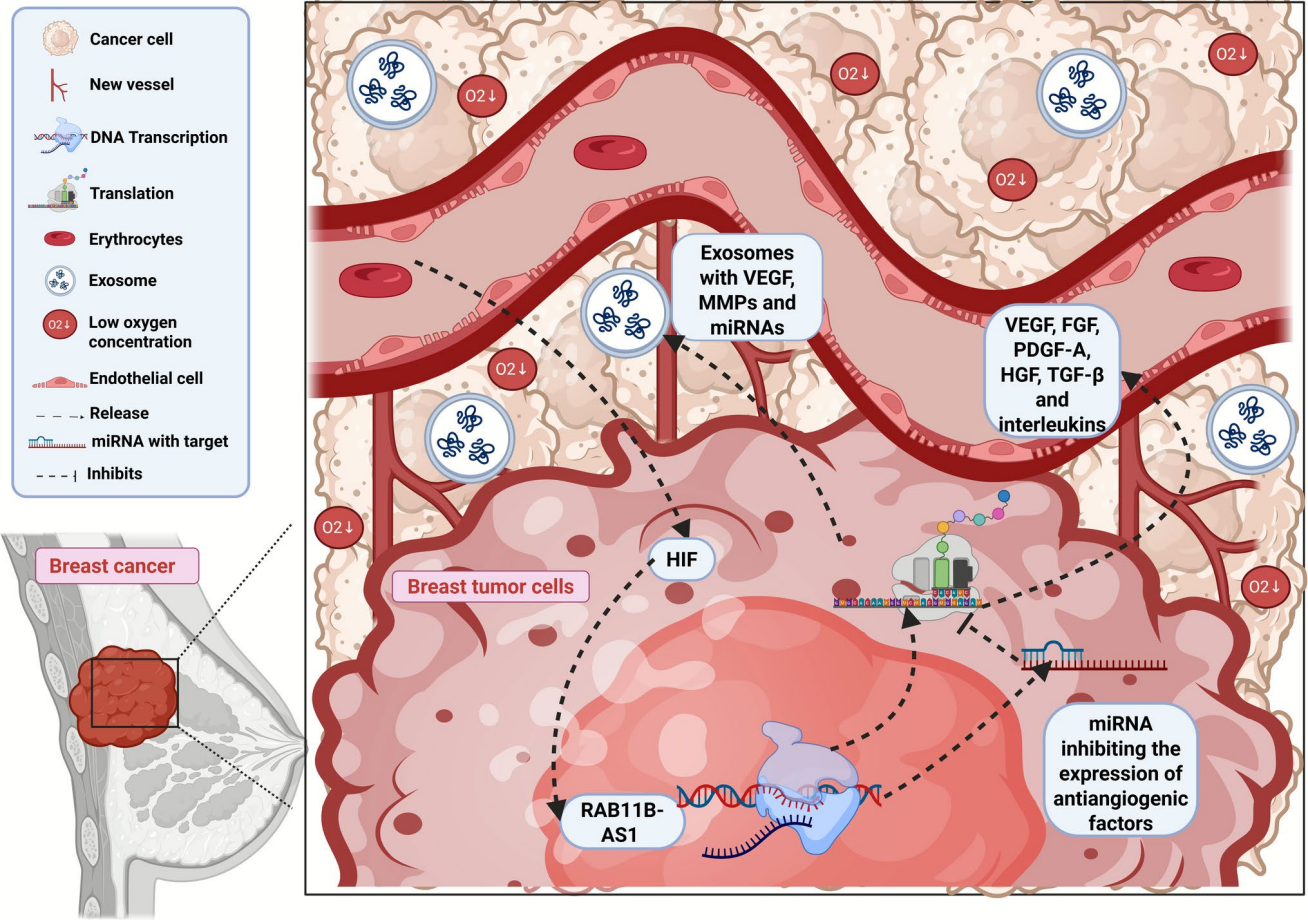
Different signals are associated to breast cancer angiogenesis. Breast tumor cells release pro-angiogenic molecules, such as VEGF, FGF, PDGF-A, HGF, TGF-β, and interleukins, triggering new vessel formation. The HIF pathway, modulation of gene expression by RAB11B-AS1 and miRNAs, and secretion of exosomes also play key roles in this process. These interactions favor the stimulation of new blood vessel formation, in addition to promoting the migration and invasion of breast tumor cells. FGF: Fibroblast Growth Factor; HIF: Hypoxia-Inducible Factor; HGF: Hepatocyte Growth Factor; PDGF-A: Platelet-Derived Growth Factor A; TGF-β: Transforming Growth Factor Beta; VEGF: Vascular Endothelial Growth Factor. Created in https://BioRender.com

Integrins are transmembrane glycoproteins that connect the cell to the ECM. In the normal tissue, integrins are a checkpoint for cell proliferation. In tumors they increase invasion, migration, and metastasis [68]. During angiogenesis they activate Akt and MAPK pathways, through ’outside-in’ signaling responsive to VEGF. This signaling initially involves integrin aggregation and recruitment of Focal Adhesion Kinase (FAK), which activates the tyrosine kinase Src and, finally, Akt/MAPK. Exosomes also trigger angiogenesis and are characterized by small extracellular vesicles (30–100 nm) that transport different molecules, such as proteins, lipids, and nucleic acids [58]. Exosomes produced by tumor cells (loaded with VEGF, MMPs and miRNAs) are rapidly internalized by endothelial cells [69, 70]. The process of induction of angiogenesis in BC is illustrated in Fig. 3. Different signaling pathways promote paracrine communication between tumor and vascular endothelial cells, generating new vasculatures, essential for tumor growth.

Given the heterogeneity of pathways and factors involved in the angiogenic process in BC, the discovery and characterization of new mechanisms are essential in the development of therapies focused on this hallmark. In this sense, purinergic signaling, known to significantly modulate vascular function, has emerged as a key point in angiogenesis, especially regarding tumors.

## Introduction to purinergic signaling

Purinergic signaling is a complex system of extracellular signaling primarily involving nucleotides and nucleosides such as ATP, ADP, UTP, UDP, and adenosine (Ado). In cells, purinergic components participate in metabolic processes crucial for enzymatic activity, energy production, and cell homeostasis. Outside the cytoplasmic environment, these components can play different roles, activating and regulating neural, cardiovascular, and immunological systems, as well as physiological and pathophysiological functions across tissues [7, 71, 72].

The extracellular function of nucleosides, such as Ado, was discovered in 1929 by Drury and Szent-Györgyi, regulating cardiac function [73]. However, the term “purinergic signaling” was first used by Geoffrey Burnstock in the early 1970s [74]. This signaling was mainly associated with neuronal interactions, as ATP was identified for the first time as a co-transmitter in sympathetic nerves. In the late 1970 s, many researchers described different families of purinergic receptors. Over the following decades, the structure of these receptors and the enzymatic family of ectonucleotidases were elucidated, consolidating the components that regulate the purinergic signaling [7, 71, 75, 76].

The effectors of purinergic signaling can be summarized by the most complex molecule (the precursor) and, in most cases, its products. ATP, for example, after being released or externalized, interacts with receptors and is processed by ectonucleotidases, losing one or two inorganic phosphates to form ADP, AMP, or Ado. Except for AMP, all these products interact with specific receptors at the plasma membrane surface [71, 77–79].

The purinergic receptors can be divided into two main groups: P2 (ATP and ADP receptors) and P1 (Ado receptors). P2 receptors can further be subdivided into P2X and P2Y, which are respectively ionotropic and metabotropic receptors. P2X receptors comprise seven different proteins that respond exclusively to ATP, and their activation leads to the influx of cations such as Na^+^, K^+^, and Ca^2+^. These receptors have two transmembrane domains and one extracellular loop, forming trimeric homomultimeric or heteromultimeric proteins. P2Y receptors can respond to adenine and uracil nucleotide agonists, triggering a range of intracellular regulatory pathways that can activate phospholipase C, or activate or inhibit adenylyl cyclase and the MAPK pathway [80, 81]. P1 receptors, in turn, form a family of G protein-coupled receptors, divided into four subtypes, A_1_, A_2a_, A_2b_ and A_3_, whose expression varies according to the tissue. Furthermore, these subtypes differ in intracellular signaling and sensitivity, as activation of A_1_ and A_3_ receptors reduces intracellular cAMP levels, while activation of A_2a_ and A_2b_ receptors promotes accumulation. Furthermore, A_1_, A_2a_ and A_3_ have greater sensitivity to Ado than A_2b_ [82]. In BC, A_2a_ and A_2b_ are overexpressed [83, 84].

Ectonucleotidases are enzymes which present their active site faced to the extracellular environment and are divided according to their structure, number of loops, and the substrate hydrolyzed. Ecto-nucleoside-triphosphate diphosphohydrolases (E-NTPDases), ecto-nucleotide pyrosphatase/phosphodiesterases (E-NPPs), alkaline ahosphatases, and ecto-5’-nucleotidase (CD73/5’-NTE). Their orchestrated function produces fewer complex molecules that can either interrupt or act antagonistically to restore system homeostasis [85].

NTPDase1 (CD39) is the primary ectonucleotidase in the vasculature, hydrolyzing ATP and ADP at a 1:1 ratio. It is expressed in both the medial and endothelial layers of blood vessels. By converting ATP to ADP and ADP to AMP, CD39 modulates vascular and platelet function, limiting thrombotic events and preserving the antithrombotic properties of the endothelium. This is significant because ADP acts as a potent platelet activator via P2Y1 and P2Y12 receptors. Therefore, CD39 effectively regulates the function of these receptors, preventing sustained activation and promoting homeostasis [7]. On the other hand, NTPDase2/CD39L1 preferentially hydrolyzes ATP over ADP, with a hydrolysis ratio of 30:1, and is predominantly found in the adventitial layer of blood vessels. Its activity increases the ADP concentration in this layer, playing a key role in vascular injury scenarios where large amounts of ADP are released [86].

CD73 hydrolyzes AMP into Ado and is mainly expressed in the vascular endothelium. Ado inhibits platelet aggregation and exerts significant anti-inflammatory effects. In healthy conditions, CD39 and CD73 form a synergistic and protective system in blood vessels. They transform a pro-thrombotic and pro-inflammatory environment rich in ATP and ADP into an anti-thrombotic and anti-inflammatory environment dominated by Ado [7, 86]. Dysregulation of ectonucleotidases is directly involved in the progression of several pathophysiological conditions, including cancer [72, 87–90].

## Purinergic signaling in cancer and the vasculature

ATP, a precursor of purinergic signaling, can also act as a damage-associated molecular pattern (DAMP), fostering migration and activating of immune system components [72]. P2 receptors control the release of cytokines and chemokines and regulate T-cells, neutrophils, macrophages, dendritic cells, B lymphocytes, NK, eosinophils, and mast cells. Their roles range from calcium mobilization and chemotaxis to protein polymerization, maturation, and degranulation [91]. In TME, ATP accumulates inside and outside the cell membrane. Inside the cell, ATP levels rise due to metabolic adaptations that make cancerous cells sustainable. Outside, ATP is converted by overexpressed ectonucleotidases, promoting an immunosuppressive environment that favors tumor progression. Furthermore, high levels of Ado can induce angiogenesis and metastasis [88, 90]. In blood vessels, the main purinergic components that participate in physiological and inflammatory functions are the receptors P2X1, P2X4, P2X7, P2Y1, P2Y2, P2Y4, P2Y6, A_1_, A_2a_, A_2b_, A_3_, and the ectonucleotidases CD39, NTPDase2, and CD73 [7, 77, 78].

**Fig. 4:**
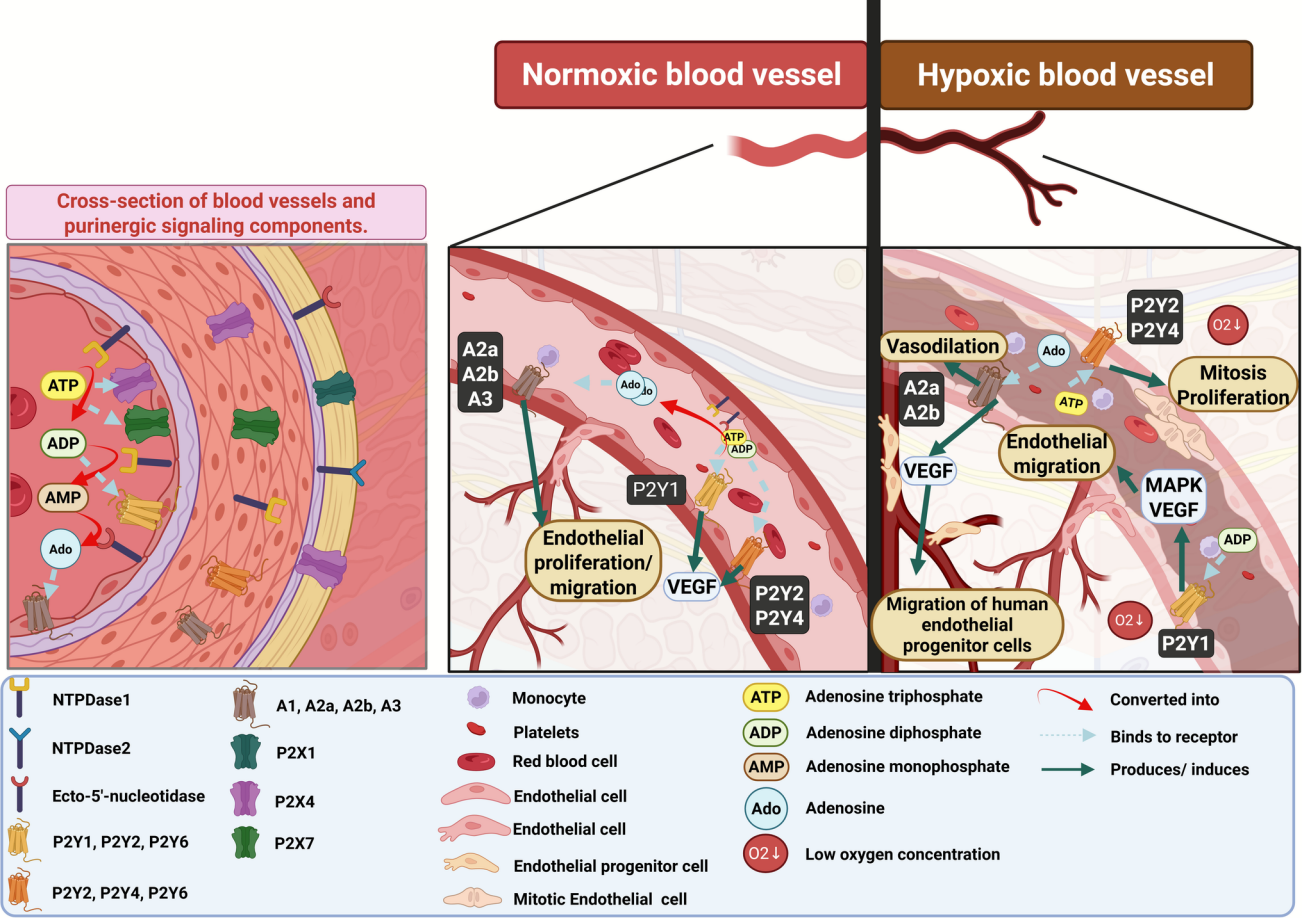
General components of purinergic signaling in blood vessels and the key mechanisms involved in angiogenesis. Under normoxic condititions, purinergic receptors activated by ATP, ADP, and adenosine regulate proliferation and migration of endothelial cells. Under hypoxic conditions, these receptors promote vasodilation, as well as the endothelial migration, proliferation and migration of human endothelial progenitor cells through MAPK and VEGF pathways, thereby facilitating angiogenesis. Additionally, under low oxygen conditions, there is an increased expression of the adenosine A2b receptor, which regulates processes such as wound healing. Ado: Adenosine; ADP: Adenosine Diphosphate; MAPK: Mitogen-Activated Protein Kinase; O₂: Molecular Oxygen; VEGF: Vascular Endothelial Growth Factor. Created in https://BioRender.com

Under physiological conditions, purines regulate vascular tone and promote smooth muscle and endothelial cell migration and proliferation, especially during angiogenesis. Ado is particularly important, as it contributes to hypoxic vasodilation and promotes angiogenesis, helping maintain tissue oxygenation. This process involves A_2b_ receptors in human microvascular endothelial cells, which modulate angiogenic factor expression and increase VEGF production. Expression of Ado receptor is also influenced by hypoxia, with A_2b_ becoming predominant in such conditions. Also, the migration of human endothelial progenitor cells, wound healing, and angiogenesis are mediated through A_2b_ and A_2a_. There are notable differences in Ado receptor responses between human micro- and macrovessel endothelial cells during hypoxia. Additionally, through P2Y1 receptors, ADP supports endothelial migration and angiogenesis by activating MAPK pathways, and VEGF-2 activation via P2Y1 receptors. UTP and ATP, mediated by P2Y2 and possibly P2Y4 receptors, also exert mitogenic and angiogenic effects on endothelial cells [7] (Fig. 4).

## Interaction of purinergic signaling with tumor angiogenesis in breast cancer

Purinergic signaling plays a key role in regulating tumor angiogenesis since extracellular nucleotides accumulate in the TME due to cell death, hypoxia, or high metabolic activity. Purinergic signaling, angiogenesis and BC are widely discussed, but their interrelationship remains little explored and poorly understood. Thus, understanding these mechanisms becomes a promising strategy for the control of these tumors, emerging new and potential therapeutic targets.

### Nucleotides and nucleosides

ATP, in addition to being an effector molecule of purinergic signaling, is the cell’s energy currency. However, there is a relationship between this nucleotide and BC growth and angiogenesis. ATP is released by tumor and blood cells during hypoxia, with a greater angiostatin-induced expression of ectopic ATP synthase, present in the inner mitochondrial membrane and the plasma membrane. It has been proven that this event is associated with tumor proliferation and angiogenesis. ATP synthase is also expressed in endothelial cells and helps maintain energy metabolism in the TME [92, 93].

In BC cells the enzyme nucleoside diphosphate kinase (sNDPK-B) is overexpressed, and is responsible for phosphorylating ADP into ATP, being regulated by the HIF pathway [94]. In TNBC, sNDPK-B is released into exosomes, which fuse with neighbored endothelial cells. This process enables a significant influx of nucleotides, activating VEGFR-2 pathway. Furthermore, endothelial cells can incorporate the enzyme into its plasma membrane, intensifying the phosphorylation process [95].

The high concentration of ATP in TME is also maintained by pannexin-1 (PANX1) and P2X7 channels [8]. Extracellular ATP acts on autocrine/paracrine, and P2Y1 and P2Y2 receptors are activated in tumor cells, stimulating the production and release of vasoactive mediators, such as nitric oxide and prostacyclins [93, 94]. Intriguingly, ATP and VEGF, together, even at low concentrations, contribute to angiogenesis. ATP is recognized as an activator of the immune system, however, when in the extracellular environment, ATP is rapidly hydrolyzed into ADP, and, subsequently, into AMP and Ado, an important immunosuppressor and proangiogenic molecule [93]. Ado production in the tumor context is hyperactivated under hypoxic conditions, due to overexpression of ectonucleotidases induced by HIF pathway [8]. This nucleotide leads to an increase in the production/secretion of VEGF, which in turn, leads to the proliferation/migration of endothelial cells, favoring angiogenesis [96]. Thrombospondin-1 production by macrophages is also controlled by Ado, modulating TME [97]. Finally, activation of A_2b_ receptors increases the production of pro-angiogenic factors and facilitates migration and tube formation by endothelial cells [98].

### Purinergic peceptors

Nucleotides and nucleosides can activate purinergic receptors on endothelial, immune, and cancer cells, influencing angiogenesis and the tumor’s overall behavior [10, 99]. P2 and P1 purinergic receptors are involved in modulating tumor angiogenesis. P2Y and P2X receptors, particularly P2Y1, P2Y12, P2X4, and P2X7 respond to ATP and other nucleotides, while P1 receptors respond to Ado. Activation of P2 receptors on endothelial cells stimulates angiogenic pathways, while Ado signaling through P1 receptors promotes immune evasion and vascular formation within the tumor [93, 99, 100].

In BC, hypoxia leads to the release of purinergic effectors and vasoactive components, which correlate with cancer progression. HIF pathway stimulates cell proliferation, differentiation, angiogenesis, and metastasis. In this context, several vasoactive molecules are secreted, such as ATP and ADP, as mentioned above. These effectors bind to P2 receptors, promoting vascular relaxation, angiogenesis, and platelet inhibition. Moreover, activation of the P2Y1 receptor transactivates VEGFR2 and promotes tubologenesis of cells in collagen matrix [101]. Similarly, previous studies performed in vitro indicated that the action of ATP on P2Y2 receptors in TNBC cells (MDA-MB231 and radiotherapy-resistant MDA-MB231) increased the secretion of IL-1β and VEGF-A, which, as mentioned, are directly related to angiogenesis [102, 103]. In BC with luminal phenotype, P2Y2 activation increases cell migration, not proliferation, by activating the MEK (Mitogen-Activated Protein Kinase Kinase)—ERK1/2 (Extracellular Signal-Regulated Kinases 1 and 2) pathway [104]. P2 receptors enhance glycolytic activity in response to ATP, and P2X4, on the other hand, is linked to neo-angiogenesis and vessel formation. In platelets, the P2Y12 receptor triggers TGF-β production, a crucial angiogenic factor [100].

Finally, P2X7 receptor activation has also been associated with the modulation of angiogenesis through TAMs polarization and VEGF release [10, 93, 100, 105, 106]. P2X7 also regulates HIF pathway, increasing intracellular Ca^2+^ and Na^+^ levels. Its action has already been described in models of melanoma, colon and prostate cancer, controlling the secretion of IL-1β, VEGF, and microvesicles containing specific sets of miRNAs, ATP and even mitochondria [107–110]. However, the role of P2X77 in BC angiogenesis has not yet been described.

The conversion of AMP to Ado marks the activation of P1 receptors, with inhibition of immune response and cellular apoptosis and promotion of proliferation, invasion and angiogenesis in BC. Therefore, the high concentration of Ado in the TME is directly correlated with an increase in the production and secretion of VEGF [83, 84, 111]. Activation of A_1_ receptors in BC promotes the release VEGF, bFGF, and IL-6 [10]. A_2a_ receptor expressed in endothelial cells close to the tumor drives the cellular response to Ado. In fact, A_2a_ receptors antagonists can reduce vascular density and pro-angiogenic effectors in murine models [84]. A_2b_ receptors also induces the production of VEGF and pro-angiogenic cytokines in endothelial and tumor cells [112]. Finally, the action of A_3_ receptor is also associated with the promotion of angiogenesis, mainly via bFGF [82, 113].

### Ectonucleotidases

CD39 and CD73 regulate tumor angiogenesis in BC, acting together to increase Ado production in TME. Therefore their overexpression and increased hydrolysis activity are correlated with increased tumor angiogenesis and a poorer prognosis [8, 114].

The CD39 modulates nucleotide and nucleoside levels in the circulation and is mainly expressed in cells of the immune system, such as macrophages, dendritic cells, neutrophils, T and B lymphocytes and NK and in endothelial cells [115–117]. The important role of CD39 in angiogenesis was demonstrated in CD39-null mice, in which cell migration of monocytes and macrophages, endothelial cells and pericytes; and the formation of new vessels was significantly lower, when compared with the control [118]. In the tumor setting, *ENTPD1* deletion caused a reduction in tumor growth and tumor formation by inhibiting angiogenesis in an in vivo mouse model [119]. Furthermore, CD39 needs to be further evaluated as a diagnostic target in BC, once it is overexpressed in luminal subtype [120].

CD73 is correlated with a poor prognosis in BC and a higher rate of angiogenesis through Ado production [113, 121]. CD73 is involved in tumor growth, metastasis, and angiogenesis by reducing ATP and ADP availability while increasing Ado levels. Consequently, T-cell infiltration and cytotoxic functions are affected. Additionally, T-cell adhesion to endothelial cells becomes a new source of pro-angiogenic factors. In pancreatic cancer, CD73 promotes tumor growth by inducing M2 macrophage formation. Moreover, CD73 knockout models demonstrate reduced tumor growth and metastasis [10, 122].

In BC, cytokines and HIF positively regulate CD73 transcription, leading to an accumulation of Ado in the TME. In this context, dendritic cells (DCs) exposed to high Ado concentrations differentiate into adenosine-differentiated DCs, expressing angiogenic factors such as VEGF, IL-8, IL-10, COX-2, and TGF-β. Pharmacological and genetic downregulation of CD73 has shown to reduce angiogenesis in both in vitro and in vivo, downregulating VEGF, IL-6, and TGF-B [10]. Furthermore, CD73 expression in BC appears to be inversely correlated with estrogen receptor expression and response. In fact, Ado production in estrogen receptor-negative breast tumors is 104 times greater than those positive for the hormone receptor [123].

**Fig. 5:**
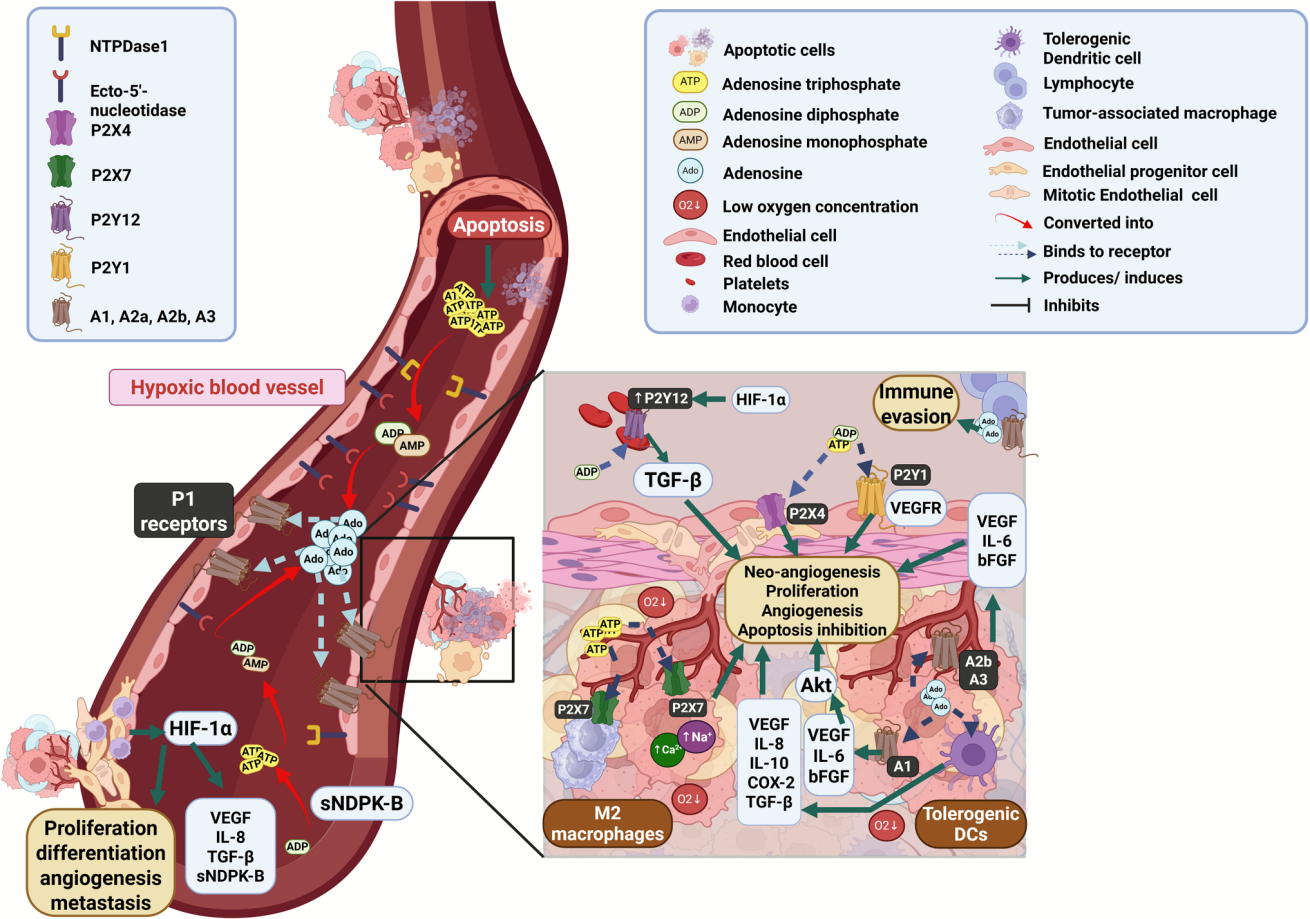
Molecular mechanisms of purinergic signaling in breast cancer angiogenesis. Purinergic effectors are released along with hypoxia-inducible factor (HIF). ATP and ADP bind to P2 receptors, promoting relaxation, differentiation, angiogenesis, and metastasis. P2Y12 platelet receptors, overexpressed by HIF, induce TGF-β release and angiogenesis. After that, both ATP and ADP, are converted into adenosine by CD39 and CD73 enzymes. Excessive adenosine, produced by overexpressed CD73, converts macrophages and dendritic cells into tumor-associated macrophages and tolerogenic dendritic cells, which release pro-angiogenic factors. Finally, activation of P1 receptors leads to immune evasion, angiogenesis, and inhibition of apoptosis. Ado: Adenosine; ADP: Adenosine Diphosphate; Akt: Protein Kinase B (PKB); AMP: Adenosine Monophosphate; ATP: Adenosine Triphosphate; bFGF: Basic Fibroblast Growth Factor; Ca^2+^: Calcium; COX-2: Cyclooxygenase-2; HIF-1α:Hypoxia-Inducible Factor 1-alpha; IL-6: Interleukin-6; IL-8:Interleukin-8; Na^+^: Sodium; sNDPK-B:Secreted Nucleoside Diphosphate Kinase B; TGF-β:Transforming Growth Factor Beta; VEGF: Vascular Endothelial Growth Factor; VEGFR: Vascular Endothelial Growth Factor Receptor. Created in https://BioRender.com

The interfaces of purinergic signaling in the angiogenesis process in BC are depicted in Fig. 5.

## Future perspectives and therapeutic innovations

For angiogenesis-targeted therapies, a greater emphasis is placed on those that explore VEGF, its receptors, and related-pathways [124]. Bevacizumab, a monoclonal antibody that binds to VEGF and inhibits its action has been used to control several solid tumors, including metastatic BC [125]. TNBC has been the main focus, and the search for synergistic compound is essential considering its complexity. Combinations with VEGFR inhibitors have been explored, with the co-administration with Receptor Tyrosine Kinase (RTK) inhibitors, EGF or FGF inhibitors or c-Met inhibitors [5, 126]. Seryl-tRNA synthetase (SerRS) was also identified as VEGFA repressor, competing with c-Myc and inducing cellular senescence and suppression of angiogenesis [127]. COX-2 has been presented as a relevant target for the control of angiogenesis in BC. An in vitro assay demonstrated that andrographolide (Andro), a diterpenoid lactone that suppresses COX-2, inhibits the proliferation of breast tumor cells (MDA-MB-231, MCF-7, T47D and MDA-MB-361) and reduces tube formation and angiogenesis using human endothelial cells (HUVECs). [128].

Regarding purinergic signaling, research is still in the early stages. The main therapeutic targets are Ado receptors. A study conducted by Cekic and collaborators demonstrated that Ado receptor inhibitors, aminophylline (non-selective) and ATL8001 (selective for A2b), reduced tumor progression, metastasis and, especially tumor angiogenesis. In this experiment, using 4T1 (metastatic BC) cell line in mice, the inhibition of P1 receptors reduced blood vessel density and VEGF levels, demonstrating that Ado signaling is a potentially relevant target in the regulation of angiogenesis in BC [129].

Ectonucleotidases are also promising. In fact, interest in understanding the modulation of these enzymes is growing, with compounds being tested at different stages of research, including in clinical phase [130]. A glycostructured monoclonal antibody against CD39 has been able to reduce angiogenesis, leading to tumor necrosis in in vitro, ex vivo and in murine cancer models ​ [131]. A tyrosine kinase inhibitor, ceritinib, has been recognized as a stable inhibitor of CD39 in in vitro assays [132]. Finally, CD73 inhibition also appears as a therapeutic potential. CD73 inhibitors can interruptecell cycle, and downregulate proliferative and pro-angiogenic factors such as COX-2, HIF-1α, STAT-3, TGF-β, IL-6, VEGF-R2, and VEGF-A in 4T1 BC mouse model [133]. Jin et al. observed that the combination of chemotherapy and photodynamic therapy (PDT) with CD73 blockade presented antimetastatic potential and increased the immune response mediated by T lymphocytes in TNBC [134]. In clinical studies, Bendell et al. demonstrated that Oleclumab, an anti-CD73 monoclonal antibody, modulates the immune response and reduces immunosuppression in different tumor types [135]. Furthermore, phase I/II clinical trials in patients with TNBC indicated a synergistic effect of this anti-CD73 when combined with anti-PD-L1 and chemotherapy (12 weekly cycles of paclitaxel and carboplatin) [130]. This CD73 inhibition also appears to be correlated with angiogenesis inhibition via VEGF reduction, as suggested by results in animal models [121].

## Conclusion

Tumor angiogenesis plays an essential role in the development of solid tumors, since rapid cell proliferation and high metabolic demand, combined with low nutrient and oxygen availability, create a hypoxic TME. This state, although initially unfavorable to the tumor, activates the HIF pathway, promoting the formation of new blood vessels to sustain transformed cells. In TME, extracellular ATP interacts with P2 receptors on endothelial and tumor cells, stimulating the release of VEGF and other pro-angiogenic factors. In addition, the ectonucleotidases CD39 and CD73 act sequentially in the hydrolysis of ATP, generating Ado, which also contributes to angiogenesis through activation of the VEGF pathway, as well as by fostering endothelial cell proliferation and migration.

Therefore, purinergic signaling pathway emerges as a promising target for therapies against angiogenesis in solid tumors, especially in BC. Here, we highlighted the potential of Ado receptor inhibitors (especially A_2b_) and ectonucleotidases CD39 and CD73 inhibitors as therapeutic options, supported in vitro, in vivo and clinical trials. However, further research is needed to fully elucidate the correlation between purinergic signaling components and angiogenesis in BC.

## Data Availability

No datasets were generated or analysed during the current study.

## Abbreviations

Ado: Adenosine
ADP: Adenosine diphosphate
ATP: Adenosine triphosphate
BC: Breast cancer
bFGF: Basic fibroblast growth factor
BRCA1/2: Breast cancer gene 1/2
CAFs: Cancer-associated fibroblasts
CCL2: C-C motif chemokine ligand 2
CD39: Ectonucleoside triphosphate diphosphohydrolase 1
CD73: Ecto-5’-nucleotidase
circRNA: Circular RNA
COX-2: Cyclooxygenase-2
CSF-1: Colony-stimulating factor 1
ECM: Extracellular matrix
E-NPPs: Ectonucleotide pyrophosphatase/phosphodiesterases
ER: Estrogen receptor
ERK1/2: Extracellular signal-regulated kinase 1/2
FAK: Focal adhesion kinase
FGF: Fibroblast growth factor
GLUT1: Glucose transporter 1
GLUT3: Glucose transporter 3
HDI: Human development index
HER2: Human epidermal growth factor receptor 2
HER2E: HER2-enriched
HIF-1α: Hypoxia-inducible factor 1-alpha
IL-1: Interleukin-1
IL-1β: Interleukin-1 beta
IL-6: Interleukin-6
Ki-67: Antigen Ki-67
LDHA: Lactate dehydrogenase A
lncRNA: Long non-coding RNA
MAPK: Mitogen-activated protein kinase
MEK: Mitogen-activated protein kinase kinase
miRNA: MicroRNA
MMPs: Matrix metalloproteinases
PANX1: Pannexin 1
PARP: Poly (ADP-ribose) polymerase
PD-1: Programmed cell death protein 1
PD-L1: Programmed death-ligand 1
PDGF: Platelet-derived growth factor
PDT: Photodynamic therapy
PHD2: Prolyl hydroxylase domain-containing protein 2
PI3K/AKT: Phosphoinositide 3-kinase/Protein kinase B
piRNA: Piwi-interacting RNA
PKM2: Pyruvate kinase M2
PR: Progesterone receptor
RTK: Receptor tyrosine kinase
SerRS: Seryl-tRNA synthetase
sNDPK-B: Secreted nucleoside diphosphate kinase B
TAM: Tumor-associated macrophage
TGF-β: Transforming growth factor-beta
TME: Tumor microenvironment
TNBC: Triple-negative breast cancer
TNF-α: Tumor necrosis factor-alpha
UDP: Uridine diphosphate
UTP: Uridine triphosphate
VEGF: Vascular endothelial growth factor

## References

[1] Ferlay J, Ervik M, Lam F, Laversanne M, Colombet M, Mery L, Piñeros M, Znaor A, Soerjomataram I, Bray F (2024) Global cancer observatory: cancer today. International Agency for Research on Cancer, Lyon, France

[2] Sung H, Ferlay J, Siegel RL (2021) Global cancer statistics 2020: GLOBOCAN estimates of incidence and mortality worldwide for 36 cancers in 185 countries. CA Cancer J Clin 71:209–249. 10.3322/caac.21660 33538338 10.3322/caac.21660

[3] Barzaman K, Karami J, Zarei Z, Hosseinzadeh A, Kazemi MH, Moradi-Kalbolandi S, Safari E, Farahmand L (2020) Breast cancer: biology, biomarkers, and treatments. Int Immunopharmacol 84:106535. 10.1016/j.intimp.2020.106535 32361569 10.1016/j.intimp.2020.106535

[4] Jiang X, Wang J, Deng X, Xiong F, Zhang S, Gong Z, Li X, Cao K, Deng H, He Y et al (2020) The role of microenvironment in tumor angiogenesis. J Exp Clin Cancer Res 39:204. 10.1186/s13046-020-01709-5 32993787 10.1186/s13046-020-01709-5 PMC7526376

[5] Elayat G, Selim A (2024) Angiogenesis in breast cancer: insights and innovations. Clin Exp Med 24:178. 10.1007/s10238-024-01446-5 39105831 10.1007/s10238-024-01446-5 PMC11303414

[6] de Heer EC, Jalving M, Harris AL (2020) HIFs, angiogenesis, and metabolism: elusive enemies in breast cancer. J Clin Invest 130:5074–5087. 10.1172/jci137552 32870818 10.1172/JCI137552 PMC7524491

[7] Burnstock G, Ralevic V (2014) Purinergic signaling and blood vessels in health and disease. Pharmacol Rev 66:102–192. 10.1124/pr.113.008029 24335194 10.1124/pr.113.008029

[8] Campos-Contreras AdR, Díaz-Muñoz M, Vázquez-Cuevas FG (2020) Purinergic signaling in the hallmarks of cancer. Cells 9:1612 32635260 10.3390/cells9071612 PMC7407645

[9] Rumjahn SM, Yokdang N, Baldwin KA, Thai J, Buxton IL (2009) Purinergic regulation of vascular endothelial growth factor signaling in angiogenesis. Br J Cancer 100:1465–1470. 10.1038/sj.bjc.6604998 19367276 10.1038/sj.bjc.6604998 PMC2694426

[10] Soleimani A, Taghizadeh E, Shahsavari S, Amini Y, Rashidpour H, Azadian E, Jafari A, Parizadeh MR, Mashayekhi K, Soukhtanloo M et al (2019) CD73; a key ectonucleotidase in the development of breast cancer: recent advances and perspectives. J Cell Physiol 234:14622–14632. 10.1002/jcp.28187 30693504 10.1002/jcp.28187

[11] Organization, WH (2024) Breast cancer. World Health Organization. http://www.who.int/news-room/fact-sheets/detail/breast-cancer. Accessed 25 Jan 2025

[12] Arnold M, Morgan E, Rumgay H, Mafra A, Singh D, Laversanne M, Vignat J, Gralow JR, Cardoso F, Siesling S et al (2022) Current and future burden of breast cancer: global statistics for 2020 and 2040. Breast (Edinburgh, Scotland) 66:15–23. 10.1016/j.breast.2022.08.010 36084384 10.1016/j.breast.2022.08.010 PMC9465273

[13] Xu Y, Gong M, Wang Y, Yang Y, Liu S, Zeng Q (2023) Global trends and forecasts of breast cancer incidence and deaths. Sci Data 10:334. 10.1038/s41597-023-02253-5 37244901 10.1038/s41597-023-02253-5 PMC10224917

[14] FOUNDATION, N.B.C (2025) Breast Cancer Facts & Stats

[15] Giaquinto AN, Miller KD (2022) Cancer statistics for African American/Black people 2022. CA Cancer J Clin 72:202–229. 10.3322/caac.21718 35143040 10.3322/caac.21718

[16] Giaquinto AN, Sung H (2024) Breast cancer statistics 2024. CA Cancer J Clin 74:477–495. 10.3322/caac.21863 39352042 10.3322/caac.21863

[17] de Araujo JB, Kerkhoff VV, de Oliveira Maciel SF, de Resende e Silva DT (2021) Targeting the purinergic pathway in breast cancer and its therapeutic applications. Purinergic Signal 17:179–200. 10.1007/s11302-020-09760-9 10.1007/s11302-020-09760-9 PMC7879595 33576905

[18] Barnard ME, Boeke CE, Tamimi RM (2015) Established breast cancer risk factors and risk of intrinsic tumor subtypes. Biochim Biophys Acta (BBA) - Rev Cancer 1856:73–85. 10.1016/j.bbcan.2015.06.002 10.1016/j.bbcan.2015.06.002 26071880

[19] Zannetti A (2023) Breast cancer: from pathophysiology to novel therapeutic approaches 2.0. Int J Mol Sci. 10.3390/ijms24032542 36768866 10.3390/ijms24032542 PMC9916418

[20] Yin L, Duan JJ, Bian XW, Yu SC (2020) Triple-negative breast cancer molecular subtyping and treatment progress. Breast Cancer Res 22:61. 10.1186/s13058-020-01296-5 32517735 10.1186/s13058-020-01296-5 PMC7285581

[21] Li J, Chen Z, Su K, Zeng J (2015) Clinicopathological classification and traditional prognostic indicators of breast cancer. Int J Clin Exp Pathol 8:8500–8505 26339424 PMC4555752

[22] Stathopoulos GP, Malamos NA, Markopoulos C, Polychronis A, Armakolas A, Rigatos S, Yannopoulou A, Kaparelou M, Antoniou P (2014) The role of Ki-67 in the proliferation and prognosis of breast cancer molecular classification subtypes. Anticancer Drugs 25:950–957. 10.1097/cad.0000000000000123 24949917 10.1097/CAD.0000000000000123 PMC4162382

[23] Höller A, Nguyen-Sträuli BD (2023) Diagnostic and prognostic biomarkers of luminal breast cancer: where are we now? Breast Cancer Targets Ther 15:525–540. 10.2147/bctt.s340741 10.2147/BCTT.S340741 PMC10392911 37533589

[24] Viale G, Hanlon Newell AE, Walker E, Harlow G (2019) Ki-67 (30-9) scoring and differentiation of luminal A- and luminal B-like breast cancer subtypes. Breast Cancer Res Treat 178:451–458. 10.1007/s10549-019-05402-w 31422497 10.1007/s10549-019-05402-w PMC6797656

[25] Yuan P, Xu B, Wang C, Zhang C, Sun M, Yuan L (2016) Ki-67 expression in luminal type breast cancer and its association with the clinicopathology of the cancer. Oncol Lett 11:2101–2105. 10.3892/ol.2016.4199 26998129 10.3892/ol.2016.4199 PMC4777879

[26] Prat A, Pineda E, Adamo B, Galván P, Fernández A, Gaba L, Díez M, Viladot M, Arance A, Muñoz M (2015) Clinical implications of the intrinsic molecular subtypes of breast cancer. Breast (Edinburgh, Scotland) 24(Suppl 2):S26-35. 10.1016/j.breast.2015.07.008 26253814 10.1016/j.breast.2015.07.008

[27] Swain SM, Shastry M (2023) Targeting HER2-positive breast cancer: advances and future directions. Nat Rev Drug Discov 22:101–126. 10.1038/s41573-022-00579-0 36344672 10.1038/s41573-022-00579-0 PMC9640784

[28] Kay C, Martínez-Pérez C (2021) Current trends in the treatment of HR+/HER2+ breast cancer. Future Oncol 17:1665–1681. 10.2217/fon-2020-0504 33726508 10.2217/fon-2020-0504

[29] Derakhshan F, Reis-Filho JS (2022) Pathogenesis of triple-negative breast cancer. Annu Rev Pathol Mech Dis 17:181–204. 10.1146/annurev-pathol-042420-093238 10.1146/annurev-pathol-042420-093238 PMC9231507 35073169

[30] Li Y, Zhang H, Merkher Y, Chen L, Liu N, Leonov S, Chen Y (2022) Recent advances in therapeutic strategies for triple-negative breast cancer. J Hematol Oncol 15:121. 10.1186/s13045-022-01341-0 36038913 10.1186/s13045-022-01341-0 PMC9422136

[31] Leon-Ferre RA, Goetz MP (2023) Advances in systemic therapies for triple negative breast cancer. BMJ 381:e071674. 10.1136/bmj-2022-071674 37253507 10.1136/bmj-2022-071674

[32] Perou CM, Sørlie T, Eisen MB, van de Rijn M, Jeffrey SS, Rees CA, Pollack JR, Ross DT, Johnsen H, Akslen LA et al (2000) Molecular portraits of human breast tumours. Nature 406:747–752. 10.1038/35021093 10963602 10.1038/35021093

[33] Network, C.G.A. (2012) Comprehensive molecular portraits of human breast tumours. Nature 490:61–70. 10.1038/nature11412 23000897 10.1038/nature11412 PMC3465532

[34] Parise CA, Caggiano V (2014) Breast cancer survival defined by the ER/PR/HER2 subtypes and a surrogate classification according to tumor grade and immunohistochemical biomarkers. J Cancer Epidemiol 2014:469251. 10.1155/2014/469251 24955090 10.1155/2014/469251 PMC4058253

[35] da Silva FC, Brandao DC, Ferreira EA, Siqueira RP, Ferreira HSV, Da Silva Filho AA, Araujo TG (2023) Tailoring potential natural compounds for the treatment of luminal breast cancer. Pharmaceuticals (Basel, Switzerland). 10.3390/ph16101466 37895937 10.3390/ph16101466 PMC10610388

[36] Slamon DJ, Leyland-Jones B, Shak S, Fuchs H, Paton V, Bajamonde A, Fleming T, Eiermann W, Wolter J, Pegram M et al (2001) Use of chemotherapy plus a monoclonal antibody against HER2 for metastatic breast cancer that overexpresses HER2. N Engl J Med 344:783–792. 10.1056/nejm200103153441101 11248153 10.1056/NEJM200103153441101

[37] Schmid P, Adams S, Rugo HS, Schneeweiss A, Barrios CH, Iwata H, Diéras V, Hegg R, Im SA, Shaw Wright G et al (2018) Atezolizumab and Nab-Paclitaxel in advanced triple-negative breast cancer. N Engl J Med 379:2108–2121. 10.1056/NEJMoa1809615 30345906 10.1056/NEJMoa1809615

[38] Domínguez-Cejudo MA, Gil-Torralvo A, Cejuela M (2023) Targeting the tumor microenvironment in breast cancer: prognostic and predictive significance and therapeutic opportunities. Int J Mol Sci. 10.3390/ijms242316771 38069096 10.3390/ijms242316771 PMC10706312

[39] Wilson BE, Gorrini C, Cescon DW (2022) Breast cancer immune microenvironment: from pre-clinical models to clinical therapies. Breast Cancer Res Treat 191:257–267. 10.1007/s10549-021-06431-0 10.1007/s10549-021-06431-0 34731350

[40] Khosravi GR, Mostafavi S, Bastan S, Ebrahimi N, Gharibvand RS, Eskandari N (2024) Immunologic tumor microenvironment modulators for turning cold tumors hot. Cancer Commun 44:521–553. 10.1002/cac2.12539 10.1002/cac2.12539 PMC11110955 38551889

[41] Benvenuto M, Focaccetti C (2024) Tumor microenvironment: cellular interaction and metabolic adaptations. Int J Mol Sci. 10.3390/ijms25073642 38612452 10.3390/ijms25073642 PMC11011721

[42] Jiang X, Shapiro DJ (2014) The immune system and inflammation in breast cancer. Mol Cell Endocrinol 382:673–682. 10.1016/j.mce.2013.06.003 23791814 10.1016/j.mce.2013.06.003 PMC4919022

[43] Niu T, Zhou F (2023) Inflammation and tumor microenvironment. Zhong Nan Da Xue Xue Bao Yi Xue Ban = J Cent South Univ Med Sci 48:1899–1913. 10.11817/j.issn.1672-7347.2023.230231 10.11817/j.issn.1672-7347.2023.230231 PMC10930746 38448384

[44] Mantovani A, Marchesi F, Porta C, Sica A, Allavena P (2007) Inflammation and cancer: breast cancer as a prototype. Breast (Edinburgh, Scotland) 16(2):S27-33. 10.1016/j.breast.2007.07.013 17764938 10.1016/j.breast.2007.07.013

[45] Murray JI, West NR, Murphy LC, Watson PH (2015) Intratumoural inflammation and endocrine resistance in breast cancer. Endocr Relat Cancer 22:R51-67. 10.1530/erc-14-0096 25404688 10.1530/ERC-14-0096

[46] Otterlei Fjørtoft M, Huse K, Rye IH (2024) The tumor immune microenvironment in breast cancer progression. Acta Oncol (Stockholm, Sweden) 63:359–367. 10.2340/1651-226x.2024.33008 10.2340/1651-226X.2024.33008 PMC11332517 38779867

[47] Deepak KGK, Vempati R, Nagaraju GP, Dasari VR, S N, Rao DN, Malla RR (2020) Tumor microenvironment: challenges and opportunities in targeting metastasis of triple negative breast cancer. Pharmacol Res 153:104683. 10.1016/j.phrs.2020.104683 32050092 10.1016/j.phrs.2020.104683

[48] Gehmert S, Lehoczky G, Loibl M, Jung F, Prantl L, Gehmert S (2020) Interaction between extracellular cancer matrix and stromal breast cells. Clin Hemorheol Microcirc 74:45–52. 10.3233/ch-199234 31796667 10.3233/CH-199234

[49] Mittal S, Brown NJ, Holen I (2018) The breast tumor microenvironment: role in cancer development, progression and response to therapy. Expert Rev Mol Diagn 18:227–243. 10.1080/14737159.2018.1439382 29424261 10.1080/14737159.2018.1439382

[50] Insua-Rodríguez J, Oskarsson T (2016) The extracellular matrix in breast cancer. Adv Drug Deliv Rev 97:41–55. 10.1016/j.addr.2015.12.017 26743193 10.1016/j.addr.2015.12.017

[51] Tufail M (2023) Unlocking the potential of the tumor microenvironment for cancer therapy. Pathol Res Pract 251:154846. 10.1016/j.prp.2023.154846 37837860 10.1016/j.prp.2023.154846

[52] Izadpanah A, Willingham K, Chandrasekar B, Alt EU, Izadpanah R (2023) Unfolded protein response and angiogenesis in malignancies. Biochim Biophys Acta (BBA) - Rev Cancer 1878:188839. 10.1016/j.bbcan.2022.188839 10.1016/j.bbcan.2022.188839 PMC10167724 36414127

[53] Ma S, Zhao Y, Lee WC, Ong LT, Lee PL, Jiang Z, Oguz G, Niu Z, Liu M, Goh JY et al (2022) Hypoxia induces HIF1α-dependent epigenetic vulnerability in triple negative breast cancer to confer immune effector dysfunction and resistance to anti-PD-1 immunotherapy. Nat Commun 13:4118. 10.1038/s41467-022-31764-9 35840558 10.1038/s41467-022-31764-9 PMC9287350

[54] Sobierajska K, Ciszewski WM, Sacewicz-Hofman I, Niewiarowska J (2020) Endothelial cells in the tumor microenvironment. Adv Exp Med Biol 1234:71–86. 10.1007/978-3-030-37184-5_6 32040856 10.1007/978-3-030-37184-5_6

[55] Anderson NM, Simon MC (2020) The tumor microenvironment. Curr Biol 30:R921-r925. 10.1016/j.cub.2020.06.081 32810447 10.1016/j.cub.2020.06.081 PMC8194051

[56] Potente M, Gerhardt H, Carmeliet P (2011) Basic and therapeutic aspects of angiogenesis. Cell 146:873–887. 10.1016/j.cell.2011.08.039 21925313 10.1016/j.cell.2011.08.039

[57] Bhat SM, Badiger VA, Vasishta S, Chakraborty J, Prasad S, Ghosh S, Joshi MB (2021) 3D tumor angiogenesis models: recent advances and challenges. J Cancer Res Clin Oncol 147:3477–3494. 10.1007/s00432-021-03814-0 34613483 10.1007/s00432-021-03814-0 PMC8557138

[58] Viallard C, Larrivée B (2017) Tumor angiogenesis and vascular normalization: alternative therapeutic targets. Angiogenesis 20:409–426. 10.1007/s10456-017-9562-9 28660302 10.1007/s10456-017-9562-9

[59] Kim J (2019) Pericytes in breast cancer. Adv Exp Med Biol 1147:93–107. 10.1007/978-3-030-16908-4_3 31147873 10.1007/978-3-030-16908-4_3

[60] Li T, Kang G, Wang T, Huang H (2018) Tumor angiogenesis and anti-angiogenic gene therapy for cancer. Oncol Lett 16:687–702. 10.3892/ol.2018.8733 29963134 10.3892/ol.2018.8733 PMC6019900

[61] Lugano R, Ramachandran M, Dimberg A (2020) Tumor angiogenesis: causes, consequences, challenges and opportunities. Cell Mol Life Sci 77:1745–1770. 10.1007/s00018-019-03351-7 31690961 10.1007/s00018-019-03351-7 PMC7190605

[62] Zhou Z, Yao H, Hu H (2017) Disrupting tumor angiogenesis and “the hunger games” for breast cancer. Adv Exp Med Biol 1026:171–195. 10.1007/978-981-10-6020-5_8 29282684 10.1007/978-981-10-6020-5_8

[63] Badodekar N, Sharma A, Patil V, Telang G (2021) Angiogenesis induction in breast cancer: a paracrine paradigm. Cell Biochem Funct 39:860–873. 10.1002/cbf.3663 34505714 10.1002/cbf.3663

[64] Varinska L, Gal P, Mojzisova G, Mirossay L, Mojzis J (2015) Soy and breast cancer: focus on angiogenesis. Int J Mol Sci 16:11728–11749. 10.3390/ijms160511728 26006245 10.3390/ijms160511728 PMC4463727

[65] Yan H, Bu P (2021) Non-coding RNA in cancer. Essays Biochem 65:625–639. 10.1042/ebc20200032 33860799 10.1042/EBC20200032 PMC8564738

[66] Annese T, Tamma R, De Giorgis M, Ribatti D (2020) MicroRNAs biogenesis, functions and role in tumor angiogenesis. Front Oncol 10:581007. 10.3389/fonc.2020.581007 33330058 10.3389/fonc.2020.581007 PMC7729128

[67] Liu R, Yu Y, Wang Q, Zhao Q, Yao Y, Sun M, Zhuang J, Sun C, Qi Y (2024) Interactions between hedgehog signaling pathway and the complex tumor microenvironment in breast cancer: current knowledge and therapeutic promises. Cell Commun Signal 22:432. 10.1186/s12964-024-01812-6 39252010 10.1186/s12964-024-01812-6 PMC11382420

[68] Yousefi H, Vatanmakanian M, Mahdiannasser M, Mashouri L, Alahari NV, Monjezi MR, Ilbeigi S (2021) Understanding the role of integrins in breast cancer invasion, metastasis, angiogenesis, and drug resistance. Oncogene 40:1043–1063. 10.1038/s41388-020-01588-2 33420366 10.1038/s41388-020-01588-2

[69] Jia Y, Chen Y, Wang Q, Jayasinghe U, Luo X, Wei Q, Wang J, Xiong H, Chen C, Xu B et al (2017) Exosome: emerging biomarker in breast cancer. Oncotarget 8:41717–41733. 10.18632/oncotarget.16684 28402944 10.18632/oncotarget.16684 PMC5522217

[70] Olejarz W, Kubiak-Tomaszewska G, Chrzanowska A, Lorenc T (2020) Exosomes in angiogenesis and anti-angiogenic therapy in cancers. Int J Mol Sci. 10.3390/ijms21165840 32823989 10.3390/ijms21165840 PMC7461570

[71] Burnstock G (2017) Purinergic signaling in the cardiovascular system. Circ Res 120:207–228. 10.1161/circresaha.116.309726 28057794 10.1161/CIRCRESAHA.116.309726

[72] Yuan X, Ferrari D, Mills T, Wang Y, Czopik A, Doursout MF, Evans SE, Idzko M, Eltzschig HK (2021) Editorial: purinergic signaling and inflammation. Front Immunol 12:699069. 10.3389/fimmu.2021.699069 34093597 10.3389/fimmu.2021.699069 PMC8170313

[73] Drury AN, Szent-Györgyi A (1929) The physiological activity of adenine compounds with especial reference to their action upon the mammalian heart. J Physiol 68:213–237. 10.1113/jphysiol.1929.sp002608 16994064 10.1113/jphysiol.1929.sp002608 PMC1402863

[74] Burnstock G (1977) The purinergic nerve hypothesis. Ciba Foundation symposium. 48:295–314. 10.1002/9780470720301.ch17 10.1002/9780470720301.ch17 24531

[75] Ribeiro JA, Cunha RA, Correia-de-Sá P, Sebastião AM (1996) Purinergic regulation of acetylcholine release. Prog Brain Res 109:231–241. 10.1016/s0079-6123(08)62107-x 9009712 10.1016/s0079-6123(08)62107-x

[76] Theobald RJ Jr (1995) Purinergic and cholinergic components of bladder contractility and flow. Life Sci 56:445–454. 10.1016/0024-3205(94)00909-0 7830505 10.1016/0024-3205(94)00909-0

[77] Ralevic V, Dunn WR (2015) Purinergic transmission in blood vessels. Auton Neurosci 191:48–66. 10.1016/j.autneu.2015.04.007 26004513 10.1016/j.autneu.2015.04.007

[78] Aslam M, Gündüz D, Troidl C, Heger J, Hamm CW, Schulz R (2021) Purinergic regulation of endothelial barrier function. Int J Mol Sci. 10.3390/ijms22031207 33530557 10.3390/ijms22031207 PMC7865261

[79] Zimmermann H (2001) Ectonucleotidases: some recent developments and a note on nomenclature. Drug Dev Res 52:44–56

[80] Mahmood A, Iqbal J (2022) Purinergic receptors modulators: an emerging pharmacological tool for disease management. Med Res Rev 42:1661–1703. 10.1002/med.21888 35561109 10.1002/med.21888

[81] Malec D (1996) Purinergic receptors. Pol J Pharmacol 48:457–465 9112687

[82] Koszałka P, Gołuńska M, Urban A, Stasiłojć G, Stanisławowski M, Majewski M, Składanowski AC, Bigda J (2016) Specific activation of A3, A2A and A1 adenosine receptors in CD73-knockout mice affects B16F10 melanoma growth, neovascularization, angiogenesis and macrophage infiltration. PLoS ONE 11:e0151420. 10.1371/journal.pone.0151420 26964090 10.1371/journal.pone.0151420 PMC4786137

[83] McNeill SM, Baltos JA, White PJ, May LT (2021) Biased agonism at adenosine receptors. Cell Signal 82:109954. 10.1016/j.cellsig.2021.109954 33610717 10.1016/j.cellsig.2021.109954

[84] Mahdizadeh M, Heydari N, Shafiei A, Akbari H, Jafari SM (2024) Adenosine receptors in breast cancer. Mol Biol Rep 51:464. 10.1007/s11033-024-09382-z 38551734 10.1007/s11033-024-09382-z

[85] Haas CB, Lovászi M (2021) Ectonucleotidases in inflammation, immunity, and cancer. J Immunol 206:1983–1990. 10.4049/jimmunol.2001342 33879578 10.4049/jimmunol.2001342 PMC10037530

[86] Morello S, Caiazzo E (2021) Thrombo-inflammation: a focus on NTPDase1/CD39. Cells. 10.3390/cells10092223 34571872 10.3390/cells10092223 PMC8469976

[87] Robson SC, Sévigny J, Zimmermann H (2006) The E-NTPDase family of ectonucleotidases: structure function relationships and pathophysiological significance. Purinergic Signal 2:409–430. 10.1007/s11302-006-9003-5 18404480 10.1007/s11302-006-9003-5 PMC2254478

[88] Nuñez-Rios JD, Ulrich H, Díaz-Muñoz M, Lameu C, Vázquez-Cuevas FG (2023) Purinergic system in cancer stem cells. Purinergic Signal. 10.1007/s11302-023-09976-5 37966629 10.1007/s11302-023-09976-5 PMC11904000

[89] Glaser T, Cappellari AR, Pillat MM, Iser IC, Wink MR, Battastini AM, Ulrich H (2012) Perspectives of purinergic signaling in stem cell differentiation and tissue regeneration. Purinergic Signal 8:523–537. 10.1007/s11302-011-9282-3 22143354 10.1007/s11302-011-9282-3 PMC3360089

[90] Pfaffenzeller MS, Franciosi MLM (2020) Purinergic signaling and tumor microenvironment in cervical cancer. Purinergic Signal 16:123–135. 10.1007/s11302-020-09693-3 32170538 10.1007/s11302-020-09693-3 PMC7166227

[91] Jacob F, Pérez Novo C, Bachert C, Van Crombruggen K (2013) Purinergic signaling in inflammatory cells: P2 receptor expression, functional effects, and modulation of inflammatory responses. Purinergic Signal 9:285–306. 10.1007/s11302-013-9357-4 23404828 10.1007/s11302-013-9357-4 PMC3757148

[92] Wang T, Ma F, Qian HL (2021) Defueling the cancer: ATP synthase as an emerging target in cancer therapy. Mol Ther Oncolytics 23:82–95. 10.1016/j.omto.2021.08.015 34703878 10.1016/j.omto.2021.08.015 PMC8517097

[93] Buxton IL, Yokdang N, Matz RM (2010) Purinergic mechanisms in breast cancer support intravasation, extravasation and angiogenesis. Cancer Lett 291:131–141. 10.1016/j.canlet.2009.09.021 19926395 10.1016/j.canlet.2009.09.021 PMC2849889

[94] Rumjahn SM, Javed MA, Wong N, Law WE, Buxton IL (2007) Purinergic regulation of angiogenesis by human breast carcinoma-secreted nucleoside diphosphate kinase. Br J Cancer 97:1372–1380. 10.1038/sj.bjc.6604019 17940513 10.1038/sj.bjc.6604019 PMC2360243

[95] Duan S, Nordmeier S, Byrnes AE, Buxton ILO (2021) Extracellular vesicle-mediated purinergic signaling contributes to host microenvironment plasticity and metastasis in triple negative breast cancer. Int J Mol Sci. 10.3390/ijms22020597 33435297 10.3390/ijms22020597 PMC7827112

[96] Masjedi A, Ahmadi A, Ghani S, Malakotikhah F, Nabi Afjadi M, Irandoust M, Karoon Kiani F, Heydarzadeh Asl S, Atyabi F, Hassannia H et al (2020) Silencing adenosine A2a receptor enhances dendritic cell-based cancer immunotherapy. Nanomed Nanotechnol Biol Med 29:102240. 10.1016/j.nano.2020.102240 10.1016/j.nano.2020.102240 32553948

[97] Ernens I, Bousquenaud M, Lenoir B, Devaux Y, Wagner DR (2015) Adenosine stimulates angiogenesis by up-regulating production of thrombospondin-1 by macrophages. J Leukoc Biol 97:9–18. 10.1189/jlb.3HI0514-249RR 25387836 10.1189/jlb.3HI0514-249RR

[98] Ludwig N, Yerneni SS, Azambuja JH, Gillespie DG, Menshikova EV, Jackson EK, Whiteside TL (2020) Tumor-derived exosomes promote angiogenesis via adenosine A(2B) receptor signaling. Angiogenesis 23:599–610. 10.1007/s10456-020-09728-8 32419057 10.1007/s10456-020-09728-8 PMC7529853

[99] Di Virgilio F, Adinolfi E (2017) Extracellular purines, purinergic receptors and tumor growth. Oncogene 36:293–303. 10.1038/onc.2016.206 27321181 10.1038/onc.2016.206 PMC5269532

[100] Aria H, Rezaei M, Nazem S, Daraei A, Nikfar G, Mansoori B, Bahmanyar M, Tavassoli A, Vakil MK, Mansoori Y (2022) Purinergic receptors are a key bottleneck in tumor metabolic reprogramming: the prime suspect in cancer therapeutic resistance. Front Immunol 13:947885. 10.3389/fimmu.2022.947885 36072596 10.3389/fimmu.2022.947885 PMC9444135

[101] Rumjahn SM, Baldwin KA, Buxton IL (2007) P2y receptor-mediated angiogenesis via vascular endothelial growth factor receptor 2 signaling. Proc West Pharmacol Soc 50:58–60 18605230 PMC3056401

[102] Jin H, Kim HJ (2020) NLRC4, ASC and caspase-1 are inflammasome components that are mediated by P2Y(2)R activation in breast cancer cells. Int J Mol Sci 21:3337. 10.3390/ijms21093337 10.3390/ijms21093337 PMC7246622 32397236

[103] Yokdang N, Tellez JD, Tian H, Norvell J, Barsky SH, Valencik M, Buxton IL (2011) A role for nucleotides in support of breast cancer angiogenesis: heterologous receptor signalling. Br J Cancer 104:1628–1640. 10.1038/bjc.2011.134 21505453 10.1038/bjc.2011.134 PMC3101911

[104] Chadet S, Jelassi B, Wannous R, Angoulvant D, Chevalier S, Besson P, Roger S (2014) The activation of P2Y2 receptors increases MCF-7 breast cancer cells migration through the MEK-ERK1/2 signalling pathway. Carcinogenesis 35:1238–1247. 10.1093/carcin/bgt493 24390819 10.1093/carcin/bgt493

[105] Salahuddin MM, Omran GA (2021) Effect of regorafenib on P2X7 receptor expression and different oncogenic signaling pathways in a human breast cancer cell line: a potential of new insight of the antitumor effects of regorafenib. Curr Issues Mol Biol 43:2199–2209. 10.3390/cimb43030154 34940128 10.3390/cimb43030154 PMC8929109

[106] Hill LM, Gavala ML, Lenertz LY, Bertics PJ (2010) Extracellular ATP may contribute to tissue repair by rapidly stimulating purinergic receptor X7-dependent vascular endothelial growth factor release from primary human monocytes. J Immunol 185(5):3028–3034. 10.4049/jimmunol.1001298 20668222 10.4049/jimmunol.1001298 PMC3156583

[107] Pegoraro A, De Marchi E, Ferracin M (2021) P2X7 promotes metastatic spreading and triggers release of miRNA-containing exosomes and microvesicles from melanoma cells. Cell Death Dis 12:1088. 10.1038/s41419-021-04378-0 34789738 10.1038/s41419-021-04378-0 PMC8599616

[108] Vultaggio-Poma V, Falzoni S, Chiozzi P, Sarti AC, Adinolfi E, Giuliani AL, Sánchez-Melgar A, Boldrini P, Zanoni M, Tesei A et al (2022) Extracellular ATP is increased by release of ATP-loaded microparticles triggered by nutrient deprivation. Theranostics 12:859–874. 10.7150/thno.66274 34976217 10.7150/thno.66274 PMC8692914

[109] Adinolfi E, Capece M, Franceschini A, Falzoni S, Giuliani AL, Rotondo A, Sarti AC, Bonora M, Syberg S, Corigliano D et al (2015) Accelerated tumor progression in mice lacking the ATP receptor P2X7. Cancer Res 75:635–644. 10.1158/0008-5472.can-14-1259 25542861 10.1158/0008-5472.CAN-14-1259

[110] De Marchi E, Pegoraro A, Turiello R, Di Virgilio F, Morello S, Adinolfi E (2022) A2A receptor contributes to tumor progression in P2X7 null mice. Front Cell Dev Biol 10:876510. 10.3389/fcell.2022.876510 35663396 10.3389/fcell.2022.876510 PMC9159855

[111] Panjehpour M, Karami-Tehrani F (2007) Adenosine modulates cell growth in the human breast cancer cells via adenosine receptors. Oncol Res 16:575–585. 10.3727/000000007783629981 18351132 10.3727/000000007783629981

[112] Gao ZG, Jacobson KA (2019) A(2B) adenosine receptor and cancer. Int J Mol Sci 20:5139. 10.3390/ijms20205139 10.3390/ijms20205139 PMC6829478 31627281

[113] Haskologlu IC, Erdag E, Ulker D, Uludag MO, Sehirli AO, Abacioglu N (2024) Chronobiologically targeted anticancer strategy: synergistic inhibition of CD39 and CD73 with adenosine receptor agonists. Springer International Publishing, Cham, pp 1–22

[114] Antonioli L, Fornai M, Pellegrini C, D’Antongiovanni V, Turiello R, Morello S, Haskó G, Blandizzi C (2021) Adenosine signaling in the tumor microenvironment. Adv Exp Med Biol 1270:145–167. 10.1007/978-3-030-47189-7_9 33123998 10.1007/978-3-030-47189-7_9

[115] Zahran AM, Rayan A (2022) Overexpression of PD-1 and CD39 in tumor-infiltrating lymphocytes compared with peripheral blood lymphocytes in triple-negative breast cancer. PLoS ONE 17:e0262650. 10.1371/journal.pone.0262650 35051220 10.1371/journal.pone.0262650 PMC8775239

[116] Xu S, Ma Y, Jiang X, Wang Q, Ma W (2024) CD39 transforming cancer therapy by modulating tumor microenvironment. Cancer Lett 597:217072. 10.1016/j.canlet.2024.217072 38885807 10.1016/j.canlet.2024.217072

[117] Bessoles S, Chiron A, Sarrabayrouse G, De La Grange P, Abina AM, Hacein-Bey-Abina S (2024) Erythropoietin induces tumour progression and CD39 expression on immune cells in a preclinical model of triple-negative breast cancer. Immunology 173:360–380. 10.1111/imm.13832 38953295 10.1111/imm.13832

[118] Goepfert C, Sundberg C, Sévigny J, Enjyoji K, Hoshi T, Csizmadia E, Robson S (2001) Disordered cellular migration and angiogenesis in cd39-null mice. Circulation 104:3109–3115. 10.1161/hc5001.100663 11748109 10.1161/hc5001.100663

[119] Jackson SW, Hoshi T, Wu Y, Sun X, Enjyoji K, Cszimadia E, Sundberg C, Robson SC (2007) Disordered purinergic signaling inhibits pathological angiogenesis in cd39/Entpd1-null mice. Am J Pathol 171:1395–1404. 10.2353/ajpath.2007.070190 17823293 10.2353/ajpath.2007.070190 PMC1988887

[120] Li C, Zhang L, Jin Q, Jiang H, Wu C (2023) CD39 (ENTPD1) in tumors: a potential therapeutic target and prognostic biomarker. Biomark Med 17:563–576 37713234 10.2217/bmm-2023-0202

[121] Allard B, Turcotte M, Spring K, Pommey S, Royal I, Stagg J (2014) Anti-CD73 therapy impairs tumor angiogenesis. Int J Cancer 134:1466–1473. 10.1002/ijc.28456 23982901 10.1002/ijc.28456

[122] Adinolfi E, Capece M, Amoroso F, De Marchi E, Franceschini A (2015) Emerging roles of P2X receptors in cancer. Curr Med Chem 22:878–890. 10.2174/0929867321666141012172913 25312206 10.2174/0929867321666141012172913

[123] Spychala J, Lazarowski E, Ostapkowicz A, Ayscue LH, Jin A, Mitchell BS (2004) Role of estrogen receptor in the regulation of ecto-5’-nucleotidase and adenosine in breast cancer. Clin Cancer Res 10:708–717. 10.1158/1078-0432.ccr-0811-03 14760094 10.1158/1078-0432.ccr-0811-03

[124] Wang L, Liu WQ, Broussy S, Han B, Fang H (2023) Recent advances of anti-angiogenic inhibitors targeting VEGF/VEGFR axis. Front Pharmacol 14:1307860. 10.3389/fphar.2023.1307860 38239196 10.3389/fphar.2023.1307860 PMC10794590

[125] Garcia J, Hurwitz HI, Sandler AB, Miles D, Coleman RL, Deurloo R, Chinot OL (2020) Bevacizumab (Avastin®) in cancer treatment: a review of 15 years of clinical experience and future outlook. Cancer Treat Rev 86:102017. 10.1016/j.ctrv.2020.102017 32335505 10.1016/j.ctrv.2020.102017

[126] Liu Y, Li Y, Wang Y, Lin C, Zhang D, Chen J, Ouyang L, Wu F, Zhang J, Chen L (2022) Recent progress on vascular endothelial growth factor receptor inhibitors with dual targeting capabilities for tumor therapy. J Hematol Oncol 15:89. 10.1186/s13045-022-01310-7 35799213 10.1186/s13045-022-01310-7 PMC9263050

[127] Zou G, Zhang X, Wang L, Li X, Xie T, Zhao J, Yan J, Wang L, Ye H, Jiao S et al (2020) Herb-sourced emodin inhibits angiogenesis of breast cancer by targeting VEGFA transcription. Theranostics 10:6839–6853. 10.7150/thno.43622 32550907 10.7150/thno.43622 PMC7295066

[128] Peng Y, Wang Y, Tang N, Sun D, Lan Y, Yu Z, Zhao X, Feng L, Zhang B, Jin L et al (2018) Andrographolide inhibits breast cancer through suppressing COX-2 expression and angiogenesis via inactivation of p300 signaling and VEGF pathway. J Exp Clin Cancer Res 37:248. 10.1186/s13046-018-0926-9 30314513 10.1186/s13046-018-0926-9 PMC6186120

[129] Cekic C, Sag D, Li Y, Theodorescu D, Strieter RM, Linden J (2012) Adenosine A2B receptor blockade slows growth of bladder and breast tumors. J Immunol 188(1):198–205. 10.4049/jimmunol.1101845 22116822 10.4049/jimmunol.1101845 PMC3819109

[130] Buisseret L, Loirat D, Aftimos P, Maurer C (2023) Paclitaxel plus carboplatin and durvalumab with or without oleclumab for women with previously untreated locally advanced or metastatic triple-negative breast cancer: the randomized SYNERGY phase I/II trial. Nat Commun 14:7018. 10.1038/s41467-023-42744-y 37919269 10.1038/s41467-023-42744-y PMC10622534

[131] Zhang H, Feng L, de Andrade Mello P, Mao C, Near R, Csizmadia E, Chan LL, Enjyoji K, Gao W, Zhao H et al (2022) Glycoengineered anti-CD39 promotes anticancer responses by depleting suppressive cells and inhibiting angiogenesis in tumor models. J Clin Investig 132: e157431. 10.1172/jci157431 10.1172/JCI157431 PMC9246388 35775486

[132] Schäkel L, Mirza S (2022) Protein kinase inhibitor ceritinib blocks ectonucleotidase CD39 - a promising target for cancer immunotherapy. J Immunother Cancer. 10.1136/jitc-2022-004660 35981785 10.1136/jitc-2022-004660 PMC9394215

[133] Ghalamfarsa G, Rastegari A, Atyabi F, Hassannia H, Hojjat-Farsangi M, Ghanbari A, Anvari E, Mohammadi J, Azizi G (2018) Anti-angiogenic effects of CD73-specific siRNA-loaded nanoparticles in breast cancer-bearing mice. J Cell Physiol 233:7165–7177. 10.1002/jcp.26743 29741783 10.1002/jcp.26743

[134] Jin F, Qi J, Liu D, You Y, Shu G, Du Y, Wang J, Xu X, Ying X, Ji J et al (2021) Cancer-cell-biomimetic upconversion nanoparticles combining chemo-photodynamic therapy and CD73 blockade for metastatic triple-negative breast cancer. J Control Release 337:90–104. 10.1016/j.jconrel.2021.07.021 34274385 10.1016/j.jconrel.2021.07.021

[135] Bendell J, LoRusso P, Overman M (2023) First-in-human study of oleclumab, a potent, selective anti-CD73 monoclonal antibody, alone or in combination with durvalumab in patients with advanced solid tumors. Cancer Immunol Immunother 72:2443–2458. 10.1007/s00262-023-03430-6 37016126 10.1007/s00262-023-03430-6 PMC10264501

